# A renewed glance at the Palearctic golden eagle: Genetic variation in space and time

**DOI:** 10.1002/ece3.11109

**Published:** 2024-03-10

**Authors:** Ekaterina Karabanina, Gerhardus M. J. Lansink, Suvi Ponnikas, Laura Kvist

**Affiliations:** ^1^ Ecology and Genetics Research Unit University of Oulu Oulu Finland

**Keywords:** *Aquila chrysaetos*, demographic history, microsatellites, mitochondrial DNA, phylogeography, population bottleneck

## Abstract

Anthropogenic pressures on nature have been causing population declines for centuries. Intensified persecution of apex predators, like the golden eagle, resulted in population bottlenecks during the 19th and 20th centuries. To study population genetics and demographic history of the golden eagle throughout its distribution, we collected museum samples from previously underrepresented regions, such as Russia and Central Asia. We used 12 microsatellite loci and a fragment of the mitochondrial DNA control region to re‐evaluate phylogeography of Eurasian golden eagles and study the impacts of the population bottleneck. Our results revealed a north–south genetic gradient, expressed by the difference between Mediterranean and Holarctic lineages, as well as genetically distinct Northern Europe and Central Asia and Caucasus regions. Furthermore, Northern Europe exhibited the lowest, whereas Central Asia and Caucasus had the highest genetic diversity. Although golden eagles maintained relatively high genetic diversity, we detected genetic signatures of the recent bottleneck, including reduced genetic diversity and a decline in the effective female population size around the year 1975. Our study improves the knowledge of the genetic composition of Eurasian golden eagles and highlights the importance of understanding their historical population dynamics in the face of ongoing and future conservation efforts.

## Graphical abstract

In this study, we investigated the phylogeography, demographic history, and impacts of past population bottlenecks in Eurasian golden eagles. Using microsatellite loci and mitochondrial DNA, we discovered a north–south genetic gradient where Central Asia and Caucasus was a high genetic diversity region, and Northern Europe showed the opposite trend. We also detected genetic signatures of a recent population bottleneck, including reduced genetic diversity and a decline in effective female population size around 1975, which highlight the significance of understanding historical population dynamics for conservation efforts.

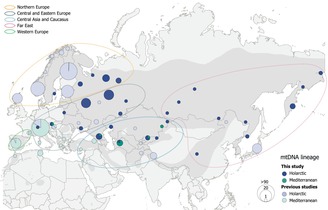

## 1 INTRODUCTION

Throughout history, humans have had a considerable impact on the distribution and viability of wild animal populations. This influence has become increasingly prominent during the last centuries due to, for example, overexploitation and habitat destruction (Dirzo et al., 2014; Newton, 2003). As a result, many populations have become small, fragmented, or even extinct (Young et al., 2016). The drastic declines in population sizes are known as population bottlenecks, which can have profound consequences for the genetic viability, adaptability, and long‐term viability of species (Frankham et al., 2010). Small population size leads to increased probability of genetic drift, loss of genetic variation, increased risks of inbreeding depression, and overall higher genetic load (Amos & Balmford, 2001; Díez‐del‐Molino et al., 2018).

Multiple factors may affect the viability of species and the outcomes of population bottlenecks, including how fast the decline happens (in generations), how long it lasts, and how many individuals are left (Amos & Balmford, 2001). Therefore, while some species are confronted with alarming rates of inbreeding and loss of genetic variation as a result of a sharp population contraction (Ewing et al., 2008), others have been thriving for hundreds and thousands of years despite small population sizes and low genetic diversity (Johnson et al., 2009; Milot et al., 2007). Additionally, a long generation time has been argued to have a profound influence on buffering the deleterious effects of bottlenecks and long‐persistent small populations by reducing the impact of genetic drift (Amos & Balmford, 2001). Studies have also emphasized the importance of population connectivity for maintaining genetic diversity (Broquet et al., 2010). Furthermore, populations on the edge of the species' distribution commonly exhibit lower genetic diversity compared to populations near the core of the distribution due to smaller effective population sizes (N_e_), reduced gene flow, and stronger geographical isolation (Eckert et al., 2008; Vucetich & Waite, 2003). Lower genetic diversity and greater differentiation of peripheral populations are the main features of the central‐marginal hypothesis (CMH), which has been confirmed in many studies (Eckert et al., 2008; Langin et al., 2017; Rönkä et al., 2019; Schwartz et al., 2003), but also contradictory evidence exits (De Kort et al., 2021; Sagarin & Gaines, 2002). Another important geographical aspect that influences genetic diversity is proximity to the past glacial refugia. Glacial refugia served as havens for species that were affected by climate cooling by providing favorable habitats that allowed populations to survive and maintain genetic variation. As a result of postglacial expansion, populations near the refugia typically show higher levels of genetic diversity, whereas populations on the expansion frontier have lower genetic diversity (Hewitt, 2000). Finally, for many species, greater intraspecific genetic variation has been found in southern regions compared to northern ones, because of a more stable environment and larger population sizes in the lower latitudes (Fonseca et al., 2023; Smith et al., 2017). For example, the lesser spotted eagle (*Clanga pomarina*) has higher mitochondrial diversity in the southern regions of its range (Väli et al., 2022). On the other hand, species more adapted to northern harsh conditions may have higher diversity in the north than in the south, as for example in the gray jay (*Perisoreus canadensis*; van Els et al., 2012).

The golden eagle (*Aquila chrysaetos*) is a long‐lived raptor with a wide Holarctic distribution (BirdLife International, 2023). As a predator of game animals and domestic livestock, the species has been heavily persecuted across Europe and North America (Watson, 2010). In addition to direct persecution, golden eagles have suffered from urban growth and forestry due to their sensitivity to anthropogenic disturbances (Watson, 2010). Altogether, these have resulted in local extinctions of golden eagles in various parts of their range (e.g., Ireland, southern Finland, and lowlands of central Europe), and in overall population declines across the Holarctic region during the 19th and 20th centuries (Bielikova et al., 2010; Nebel et al., 2015; Ollila, 2018; Starikov, 2020). Golden eagles were protected in most parts of their distribution by the end of the 20th century (Below, 2000; Sato et al., 2017; Whitfield et al., 2008). As a consequence of conservation efforts and the species' extensive range, the golden eagle is currently classified as *Least Concern* by the International Union for Conservation of Nature (IUCN) both globally, and in Europe (BirdLife International, 2023). The classification reflects the overall stability, but regional populations continue to face local threats, such as habitat destruction, human disturbances, use of lead bullets and pesticides, collisions with wind turbines, and illegal trade (D'Addario et al., 2019; Slabe et al., 2022; Watson, 2010). Unfortunately, some vast regions within the species' distribution, such as Russia and much of Asia, are lacking data on golden eagles, limiting conservation efforts. For example, only a few small‐scale scientific expeditions have been organized to collect information on breeding, ecology, and distribution of golden eagles in Russia, Kazakhstan, and Mongolia (Isaev et al., 2021; Shagdarsuren, 1964; Smelansky et al., 2020). These expeditions have revealed that the species is generally rare in many parts of Russia and Kazakhstan, and that there has been a noticeable decline in their numbers in several regions in recent times (Kazansky & Babushkin, 2021; Kerdanov & Nikolaev, 2019).

The first studies that used population genetic tools for golden eagles revealed interesting insights into their population history (Bourke et al., 2010; Judkins & van den Bussche, 2017; Naito‐Liederbach et al., 2021; Nebel et al., 2019, 2023). For example, using global golden eagle data, Nebel et al. (2015) identified two distinct mitochondrial lineages: a Mediterranean and a Holarctic. Holarctic haplotypes were found across Europe, Asia, and North America, while the Mediterranean lineage was restricted to the Mediterranean region (Judkins & van den Bussche, 2017; Nebel et al., 2015). A subsequent study using microsatellites demonstrated genetic differentiation between Northern (Norway, Finland, and Estonia) and Southern (Mediterranean and Alpine regions) Europe, with a distinct population in Scotland (Nebel et al., 2019). However, the detected nuclear differentiation was not identical to the differentiation of the mitochondrial lineages, as is often expected due to different mutation rates and thus different temporal scales of these markers. Meanwhile, genetic research on golden eagles in Asia remains sparse, with the exception of the extensive works on Japanese golden eagles (Masuda et al., 1998; Naito‐Liederbach et al., 2021; Sato et al., 2017) and a recent study in the Mongol‐Altai region (Nebel et al., 2023).

Here we aimed to improve the knowledge on phylogeography of Eurasian golden eagles by incorporating previously undersampled regions, such as Russia and Central Asia. We used a combination of mitochondrial and nuclear genetic markers to (1) re‐evaluate the population structure and genetic differences between golden eagle populations in the Palearctic. By complementing the existing data on golden eagles with samples from understudied regions of the species' distribution, we aimed to refine the eastern boundary of the Mediterranean lineage and establish the genetic relationships among golden eagles from different geographical regions. We also (2) estimated genetic diversity across the Palearctic with the assumption that in the center of the distribution and in the southern regions, the populations would harbor more genetic diversity than the northern regions and at the edges of the distribution. This assumption was raised due to the existence of both lineages in the south, the proximity of southern populations to the suggested glacial refugia around the Mediterranean Sea, and due to that many species have higher diversity in the center than at the edges of their distribution. Finally, we (3) studied demographic history and the effects of the recent population bottleneck on the genetic variation of this species. We presumed that golden eagles might show reduced genetic variation due to the population bottleneck of the 19th and 20th centuries, although this effect could be mitigated by the species' long generation time and high dispersal potential.

## 2 MATERIALS AND METHODS

### 2.1 Sampling and laboratory analyses

We collected 86 golden eagle samples from across Eurasia (Figure S1) dated between 1885 and 2017 (Table S1). The skin (*N* = 82) and a feather (*N* = 1) samples were taken from museum collections, while shed feathers (*N* = 3) were taken from captive adults. We extracted genomic DNA from skin samples (c. 0.5 × 0.5 cm pieces) with E.Z.N.A.® Tissue DNA Kit following the modified Mouse Tail Snips Protocol (Omega Bio‐Tek, USA). The volume of TL Buffer was increased to 400 μL to fully cover the tissues. To enhance the dissolving of keratin from residue feathers, we added 20 μL of dithiothreitol (DTT) to each sample before an overnight incubation. When lysis was incomplete, an extra 20 μL of protease solution was added, and the sample was vortexed and left in an incubator for additional 60–90 min. The amount of DNA wash buffer was decreased to 650 μL for both washing steps. Elution was done only once with 50 μL of Elution buffer.

Feather samples were purified with chlorine prior to extraction. First, we cut off the quill ends of feathers to Eppendorf tubes. When possible, a part of a quill with a blood clot was taken. Then, we added 10% chlorine to each sample, and vortexed and centrifuged the samples for 30 min at maximum speed. After that, we discarded the chlorine, washed the sample three times with sterile water, and left them to dry with open lids at room temperature. The subsequent DNA extraction was done the same way as with the skin samples.

A maximum of 16 samples were prepared at once to reduce the risks of cross‐contamination. Furthermore, all DNA extractions were done in a dedicated clean laboratory that was UV‐sterilized before and after working there. Contemporary feather samples were prepared in a general molecular ecology lab and subsequently extracted in the clean lab. For all samples, the DNA concentration was measured with NanoDrop (Thermo Scientific, Waltham, MA, USA), and it varied between 0.25 and 477 ng/μL (median 60.0, mean 95.5 ng/μL). All DNA extractions were stored at −20°C.

We sequenced a fragment of a mitochondrial DNA control region (mtDNA CR) with primers modGOEA_CR1L (5′‐CCCCCGTATGTATTATTGTA‐3′, Nebel et al., 2015) and GOEA_CR595H (5′‐GCAAGGTCGTAGGACTAACC‐3′ Sonsthagen et al., 2012) and genotyped golden eagles with 12 microsatellite loci: Aa02, Aa04, Aa11, Aa15, Aa26, Aa27, Aa35, Aa36, Aa39, Aa43, NVHfr142, and NVHfr206 (Table S2; Bielikova et al., 2010; Martinez‐Cruz et al., 2002; Nesje & Røed, 2000) following Kylmänen et al. (2023). We added five randomly selected golden eagles from Finland that had been genotyped and sequenced in our previous study (Kylmänen et al., 2023) to better cover the Palearctic distribution of the species.

### 2.2 Sequencing and genotyping quality

The mtDNA sequences were manually edited and aligned based on CLUSTAL W Multiple Alignment (Thompson et al., 1994) in BioEdit v.7.2.5 (Hall, 1999). We performed microsatellite genotyping of all individuals twice, and for samples with weaker amplification (20% of the duplicates), thrice. The alleles were scored with GeneMapper v.5.0. (Thermo Scientific, Waltham, MA, USA). To minimize genotyping errors and verify the fit of our markers for downstream analyses, we created consensus genotypes by selecting alleles that were consistently replicated, or by including a heterozygote when two out of three replicates showed both a homozygote and a heterozygote. When alleles in the replicates were inconsistent, the specific marker was called missing. Further, we estimated genotyping success with Microsat_errcalc (Honka & Merikanto, 2020) by calculating error, allelic dropout (ADO), and false‐allele (FA) rates. The presence of null alleles and stuttering was checked with MicroChecker 2.2.3 (Van Oosterhout et al., 2004), and null allele frequencies were calculated with FreeNA (Chapuis & Estoup, 2007). Deviations from Hardy–Weinberg equilibrium (HWE) and linkage equilibrium were tested with Genepop v.4.0.10 (Rousset, 2008) with 1000 permutations.

### 2.3 Data analyses

#### 2.3.1 Datasets

Our dataset consisted of 91 golden eagles (Table S1), of which 86 were the newly obtained samples and five were added from Kylmänen et al. (2023). This dataset is hereafter referred to as the main dataset. To present a more comprehensive overview of the species in Eurasia, we included sequences from GenBank (Table S3) for various mitochondrial analyses (Table S4; see details below). We used three grouping criteria for the downstream analyses.

**FIGURE 1:**
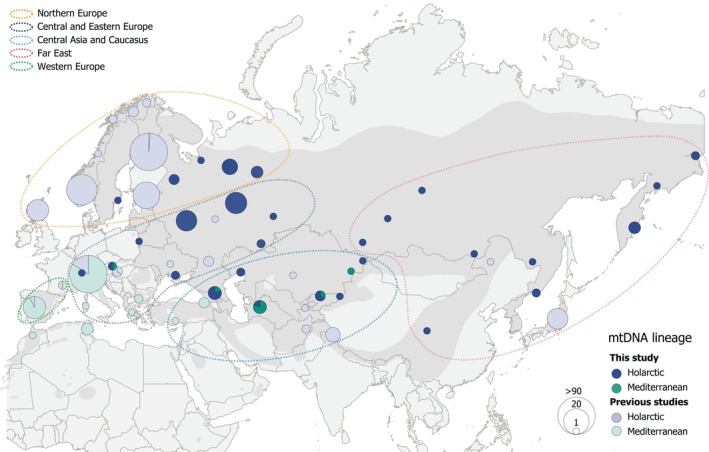
The locations of golden eagles with the Mediterranean and the Holarctic haplotypes from this (dark green and dark blue) and previous studies (light green and light blue; Kylmänen et al., 2023; Nebel et al., 2015, 2019). The size of a circle corresponds to the number of golden eagles in the area or country with either a Holarctic or a Mediterranean lineage haplotype. The ellipses outline five geographical groups used in this study. African samples from Nebel et al. (2015) are not included in the analyses because their sequences were not available. Eurasian distribution range of golden eagles is shaded with dark gray color (BirdLife International, Handbook of the Birds of the World, 2022). The map was created in QGIS 3.10 (QGIS Development Team, 2022).

First, the samples were grouped according to their geographical origin (Figure 1): (1) Northern Europe, *N* = 19 (our data) and *N* = 173 (our data + GenBank), (2) Central and Eastern Europe, *N* = 39 (our data) and *N* = 191 (our data + GenBank), (3) Central Asia and Caucasus, *N* = 18 (our data) and *N* = 22 (our data + GenBank), (4) Far East, *N* = 15 (our data) and *N* = 32 (our data + GenBank), and (5) Western Europe, *N* = 16 (only GenBank data). The latitudinal split between Northern Europe and Central and Eastern Europe was arbitrary and according to division of Russian federal districts, with samples from the Northwestern federal district assigned to Northern Europe, and samples from the Central and the Volga federal districts assigned to Central and Eastern Europe. In addition, Northern Europe included samples from Finland, Sweden, Norway, and Scotland, while Central and Eastern Europe comprised samples from Alps, Austria, Belarus, Bulgaria, Greece, Hungary, Italy, Romania, and Ukraine. Central Asia and Caucasus included samples from the Caucasus region (Russian North Caucasian federal district and Azerbaijan) and Central Asian countries (Afghanistan, Kazakhstan, Kyrgyzstan, Turkmenistan, and Uzbekistan) including Iran and Turkey. Samples collected east from the Ural Mountains, specifically from the Siberian and the Far Eastern federal districts in Russia, China, and Japan, formed the Far East group. Western Europe consisted only of GenBank sequences from Spain and non‐Alpine France. Details on sample allocation into five geographical groups are presented in Table S5.

The second grouping was done according to the mitochondrial lineages defined by Nebel et al. (2015): Mediterranean (*N* = 8 our data, *N* = 150 our data + GenBank) and Holarctic (*N* = 79 our data, *N* = 284 our data + GenBank). These lineages are distinguished by several fixed substitutions in the studied part of the mitochondrial control region and differ in their geographical distributions. The third subdivision was based on temporal groups: a Bottleneck group (until the year 1984; *N* = 67 our data, *N* = 147 our data + GenBank) and a Post‐bottleneck group (from the year 1985 onwards; *N* = 13 our data, *N* = 246 our data + GenBank). The temporal groups were chosen based on information on protective legislation and the generation time of golden eagles. We calculated golden eagle generation time using the formula described by Brown (1966) as the time taken by two adults to replace themselves: 2/(preadult survival rate × number of chicks per pair per year). The survival and reproduction success values were taken from published data for golden eagles from Finland, Sweden, and Scotland (Table S6; Ollila, 2018; Sulkava et al., 1984; Whitfield et al., 2004). Thus, the average generation length of golden eagles was 14.56 years ranging between 9.14 and 23.50 years. Since golden eagles were protected in many countries by the 1970s, by adding one generation as a buffer, we considered the year 1985 as the borderline between the two temporal groups.

#### 2.3.2 MtDNA analyses

We calculated the number of haplotypes (H), haplotype (h), and nucleotide (*π*) diversities, and Watterson's theta (*θ_W_*, from the number of segregating sites) using DnaSP v.6.12 (Rozas et al., 2017) for every group and for the entire Eurasian population. We estimated genetic diversity using newly sequenced individuals including the GenBank sequences (*N* = 434 for geographical and *N* = 393 for temporal groups; Kylmänen et al., 2023; Nebel et al., 2015, 2019). Additionally, we repeated this step using the main dataset of a longer sequence alignment (390 bp) of the newly sequenced individuals plus five Finnish golden eagles from Kylmänen et al. (2023), because it had a few additional variable sites (*N* = 87 for geographical and *N* = 77 for temporal groups). To account for different sample sizes, we performed sample size‐based rarefaction‐extrapolation for the number of haplotypes using the “iNEXT” package (Chao et al., 2014; Hsieh et al., 2016) in R v.4.0.4 (R Core Team, 2021) with 1000 bootstrap replications. The rarefaction‐extrapolation analyses were only performed for the dataset that included the GenBank sequences (*N* = 434 for geographical and mitochondrial lineage groups and *N* = 393 for temporal groups).

A median joining‐haplotype network (Bandelt et al., 1999) for the entire Holarctic region was constructed in PopART (Leigh & Bryant, 2015). For that, we used golden eagles sequenced in this study (*N* = 82) and GenBank sequences of golden eagles from Eurasia with location information (*N* = 352, Kylmänen et al., 2023; Nebel et al., 2015, 2019) and North America (*N* = 229, Craig et al., 2016; Judkins & van den Bussche, 2017; Nebel et al., 2015; Sonsthagen et al., 2012; Table S3). Haplotype and trait files for PopART were created using packages “pegas” (Paradis, 2010) and “ape” (Paradis & Schliep, 2019) in R. Haplotype frequencies from the previous studies were used as reported in Nebel et al. (2015, 2019), Craig et al. (2016), Judkins and van den Bussche (2017), and Kylmänen et al. (2023), while for Sonsthagen et al. (2012) the frequencies were unknown, and we used one sequence per haplotype.

To further investigate the genetic structure, we calculated pairwise *F*_ST_ values and performed analysis of molecular variance (AMOVA) for the three groupings in Arlequin v.3.5 (Excoffier & Lischer, 2010) with 10,000 permutations and using Kimura 2‐parameter distance model with gamma distribution (shape parameter of 0.05). Both the distance model and the shape parameter were estimated in MEGA X (Kumar et al., 2018), and the model with the lowest Bayesian Information Criterion (BIC) value was chosen. BIC was selected over Akaike Information Criterion (AIC) because it performs better in selecting the correct model for explaining the existing data (Aho et al., 2014; Chakrabarti & Ghosh, 2011). We checked for signals of population size changes with the three most powerful neutrality tests in detecting expansions (Ramírez‐Soriano et al., 2008): Tajima's D (Tajima, 1989), Fu's Fs (Fu & Li, 1993), and Ramos‐Onsins and Rozas R_2_ (Ramos‐Onsins & Rozas, 2002) in DnaSP. We also constructed mismatch distribution graphs in DnaSP as indicators of population demographic changes (Harpending, 1994). These analyses were performed using our sequences and including the GenBank data (*N* = 434 for geographical and *N* = 393 for temporal groups; Kylmänen et al., 2023; Nebel et al., 2015, 2019). Similarly, we repeated AMOVA and pairwise *F*_ST_ estimations using a longer sequence alignment (390 bp) of the main dataset (*N* = 87 for geographical and *N* = 77 for temporal groups).

To explore temporal population dynamics, we constructed a temporal haplotype network (TempNet, Prost & Anderson, 2011) with sequences from this study (*N* = 72) and Eurasian golden eagles from GenBank (*N* = 321; Kylmänen et al., 2023; Nebel et al., 2015) which had available information on the year of the sample. With the same dataset, we generated Bayesian skyline plots (BSP, Drummond et al., 2005) in BEAST 2 (Bouckaert et al., 2014) to identify possible fluctuations in effective female population sizes (N_ef_) of the total Eurasian population, and of Mediterranean and Holarctic lineages separately. We applied a strict molecular clock fixing the rate to 1 because no estimates of the clock rate for golden eagles or closely related species were available (Drummond et al., 2006). We used years of samples as tip dates and applied the Hasegawa‐Kishino‐Yano (HKY) substitution model. We estimated the substitution rates, HKY frequencies, and kappa, and chose two gamma categories with a shape parameter of 0.50 and the proportion of invariable sites of 0.86. We ran 100 million Markov Chain Monte Carlo (MCMC) with a 10% burn‐in, sampling model parameters and genealogies every 1000 iterations. After the first run, we implemented the recommended corrections to operators (Table S7) and performed multiple simultaneous runs, which were afterward combined using LogCombiner (implemented in BEAST) to achieve sufficient effective sample sizes (ESS > 200). The ESSs above 200 were achieved after eight runs for the total dataset, after six runs for the Holarctic dataset, and after two runs for the Mediterranean dataset (Table S7). Additionally, we repeated the analyses treating these groups as independent datasets, but since the results did not change, we only reported the settings described above. The Bayesian Skyline reconstruction was done in Tracer v.1.7.2 (Rambaut et al., 2018).

#### 2.3.3 Microsatellite analyses

We calculated the polymorphic information content (PIC) for each locus in Cervus 3.0.7 (Kalinowski et al., 2007) and the number of alleles (A), observed heterozygosity (H_O_), and unbiased expected heterozygosity (H_E_) in GenAlEx 6.5 (Peakall & Smouse, 2012). The allelic richness (AR) and inbreeding coefficients (F_IS_) were calculated using FSTAT 2.9.4 (Goudet, 2003). Estimates of allelic richness were based on eight diploid individuals for the four geographical groups, on six diploid individuals for the mitochondrial lineage groups, on 11 diploid individuals for the temporal dataset, and on 47 diploid individuals for the total dataset. To check whether F_IS_ significantly deviated from zero, we used the one‐sample Wilcoxon signed rank test in R. Differences in genetic diversity (A, H_O_, H_E_, F_IS_) between the mitochondrial lineage groups and between the temporal groups were tested using the Mann–Whitney *U* test (Wilcoxon rank sum test) in R. To statistically compare genetic diversity among the four geographical groups, we used the Kruskal–Wallis rank sum test in R for simultaneous comparison of the groups, and the Mann–Whitney *U* test for pairwise comparison between groups. Statistical comparisons of F_IS_ between the temporal groups and among the geographical groups were done with 11 loci: locus NVHfr206 was excluded, because expected heterozygosities in the Post‐bottleneck group and in the Northern Europe group for this locus were zero, making calculation of the inbreeding coefficient impossible. Furthermore, we used ADZE (Szpiech et al., 2008) to calculate the private allelic richness (PAR) per group.

Population structure was studied with STRUCTURE 2.3.4 (Pritchard et al., 2000), where we applied 500,000 MCMC chains with 20% burn‐in and performed 10 iterations for each of 1–5 possible clusters (K). We used the admixture ancestry model (alpha inferred from the data) with correlated allele frequencies. We ran STRUCTURE de novo (i.e., without LOCPRIOR), and using geographical groups, mitochondrial lineages, and temporal groups as LOCPRIORs. Additionally, to exclude the influence of a strong genetic differentiation of Central Asia and Caucasus and check for the existence of a substructure among the other populations, we ran STRUCTURE excluding individuals from this group. The most likely number of clusters was estimated using three methods: Evanno ΔK (Evanno et al., 2005), standard log probability test (L(K), Pritchard et al., 2000) and the Puechmaille's optimal K (Puechmaille, 2016), all implemented in Structure Selector (Li & Liu, 2018). We examined the results of all four Puechmaille's K estimators (median of medians, median of means, maximum of medians, and maximum of means), and if differences were observed, we chose the median of medians (MedMedK), because this parameter is less affected by incorrect grouping of individuals into populations and presence of migrants, and it helps to avoid overestimation, which might occur with estimators based on the maximum (MaxMeaK and MaxMedK, Puechmaille, 2016). We visualized the results of assignment with the online tool POPHELPER (Francis, 2016).

To examine the consistency of population structuring, we also applied the Discriminant analysis of principal components (DAPC, Jombart et al., 2010) implemented in “adegenet” 2.1.3 (Jombart, 2008) package in R. We performed DAPC analysis for all pre‐defined groups and with de novo grouping of individuals into clusters. For the de novo assignment we used the find. clusters() command and selected K according to the lowest BIC score. For choosing the optimal number of principal components (PCs) to retain in the discriminant analysis, we applied the cross‐validation method with 1000 permutations. In addition, we used GenePlot in the “geneplot” (McMillan & Fewster, 2017) package in R to visualize the genetic assignment of individuals. In GenePlot, we performed Principal Components Analysis (PCA) for geographical groups, log genotype probability (LGP) test for pairwise comparisons of these groups, LGP test to compare Mediterranean and Holarctic lineages, and LGP test to compare the temporal groups. For these analyses, we only included individuals with a minimum of eight genotyped loci. We calculated pairwise *F*_ST_ values between groups and performed AMOVA with number of different alleles as a distance method using 10,000 permutations in Arlequin. To test for isolation by distance (IBD) we used the Mantel test and the spatial autocorrelation test, both implemented in GenAlEx.

To additionally check for signs of population bottlenecks, we ran the Bottleneck program (Piry et al., 1999) using the two‐phase mutation model (TPM) with variance set to 30, and a proportion of SMM (stepwise mutation model) of 80% in TPM. We used the Wilcoxon sign rank test for heterozygote excess with 10,000 replications and the mode‐shift test to identify groups with signs of bottlenecks.

## 3 RESULTS

### 3.1 Sequencing and genotyping quality

We succeeded to sequence a 390 bp fragment of the mtDNA CR from 82 out of the 86 golden eagles. Four individuals were excluded due to low sequence quality. With the addition of five Finnish individuals from Kylmänen et al. (2023), we created a main mtDNA dataset of 87 golden eagles.

All 86 individuals were genotyped with 12 polymorphic loci (Table S8). Including the five Finnish individuals (Kylmänen et al., 2023), the total microsatellite dataset consisted of 91 individuals (i.e., the main dataset). Mean genotyping success was 87.6%, being the lowest for loci Aa35 (51.6%) and Aa11 (67.0%). However, since most samples were from old museum specimens that usually have lower genotyping success because of the degraded DNA (Tsai et al., 2020), and since these loci had high PIC values (0.74 and 0.59, respectively), we included them into the analyses. The number of alleles varied between 2 and 13 per locus and was on average 7.8. The error rate was 0.036 per allele and 0.078 per locus. The mean ADO rate was 0.056 and the mean FA rate was 0.022. MicroChecker suggested the presence of null alleles in loci Aa04, Aa11, Aa35, Aa36, Aa39, and NVHfr142, and stuttering in loci Aa35, Aa36, and Aa39. However, these results were not consistent when the four geographical groups were analyzed separately, indicating that the excess of homozygotes was not a genotyping artifact and was rather attributed to undetected population structure (e.g., Wahlund effect; Garnier‐Géré & Chikhi, 2013). The mean null allele frequency over all loci was 0.076. No consistent significant deviations from HWE and linkage equilibrium were observed when groups of different datasets were tested separately. Therefore, all loci were kept for the downstream analyses.

### 3.2 Spatial genetic variation

#### 3.2.1 Genetic diversity

The genetic diversity of Eurasian golden eagles is summarized in Table 1. From the dataset of 434 individuals, which included the GenBank sequences, we identified 40 haplotypes. Overall, nucleotide diversity was higher than theta (*π* = 0.0164, *θ*_*w*_ = 0.0079), and this pattern remained for Central and Eastern Europe and Central Asia and Caucasus. These two groups also had the highest nucleotide diversity. Far East exhibited the highest haplotype diversity and the highest number of haplotypes (Figure S2a), while its nucleotide diversity was relatively low. The lowest mitochondrial diversity was in Northern Europe, with notably lower nucleotide diversity compared to theta (*π* = 0.0034, *θ*_*w*_ = 0.0087). Between the lineages, each mitochondrial genetic diversity parameter was lower in the Mediterranean group than in the Holarctic group, including the number of haplotypes after the rarefaction‐extrapolation analysis (Figure S2b).

**TABLE 1:** Mitochondrial and nuclear genetic diversity of Eurasian golden eagles for geographical groups (Northern Europe, Central and Eastern Europe, Central Asia and Caucasus, Far East, and Western Europe^a^), mitochondrial lineages (Mediterranean and Holarctic), temporal groups (Bottleneck and Post‐bottleneck) further subdivided into lineages, and the total population.

Group	Mitochondrial DNA								Nuclear microsatellites						
	*N*	H	h	*π*	*θ*_*w*_ (S)	Tajima's *D*	Fu's Fs	R_2_	*N*	H_O_	H_E_	F_IS_	A	AR	PAR
Geographical group															
Northern Europe	173	15	0.574	0.0034	0.0087	−1.59 (*p* = .02)	−8.04 (*p* = .006)	0.05 (*p* = .04)	19	0.496	0.585	0.151	63	4.03	0.29
Central and Eastern Europe	191	19	0.756	0.0156	0.0100	1.49 (*p* = .94)	0.2 (*p* = .59)	0.13 (*p* = .95)	39	0.471	0.611	0.242	68	4.16	0.36
Central Asia and Caucasus	22	10	0.823	0.0177	0.0126	1.45 (*p* = .96)	−0.05 (*p* = .54)	0.19 (*p* = .95)	18	0.514	0.659	0.218	71	4.98	0.78
Far East	32	12	0.833	0.0082	0.0084	−0.06 (*p* = .55)	−3.55 (*p* = .03)	0.02 (*p* = .01)	15	0.597	0.651	0.098	58	4.21	0.39
Western Europe^a^	16	6	0.750	0.0098	0.0120	−0.71 (*p* = .27)	0.58 (*p* = .64)	0.12 (*p* = .21)	–	–	–	–	–	–	–
Mitochondrial lineage															
Mediterranean	150	13	0.600	0.0028	0.0055	−1.19 (*p* = .11)	−7.4 (*p* = .006)	0.05 (*p* = .15)	8	0.517	0.623	0.212	58	4.39	1.34
Holarctic	284	27	0.614	0.0039	0.0079	−1.26 (*p* = .08)	−22.53 (*p* < .001)	0.03 (*p* = .17)	79	0.508	0.654	0.190	86	3.81	1.08
Temporal group															
Bottleneck	147	29	0.754	0.0141	0.0121	0.46 (*p* = .74)	−7.41 (*p* = .03)	0.10 (*p* = .74)	67	0.51	0.628	0.195	88	4.86	1.08
Bottleneck Mediterranean (1857–1982)	35	9	0.620	0.0031	0.0067	−1.61 (*p* = .01)	−4.89 (*p* < .001)	0.06 (*p* = .03)	–	–	–	–	–	–	–
Bottleneck Holarctic (1817–1984)	112	20	0.611	0.0042	0.0075	−1.16 (*p* = .13)	−14.48 (*p* < .001)	0.05 (*p* = .19)	–	–	–	–	–	–	–
Post‐bottleneck	246	25	0.800	0.0174	0.0101	1.91 (*p* = .97)	−1.03 (*p* = .45)	0.14 (*p* = .98)	13	0.518	0.61	0.146	60	4.63	0.95
Post‐bottleneck Mediterranean (1989–2013)	109	7	0.598	0.0028	0.0035	−0.45 (*p* = .39)	−1.45 (*p* = .24)	0.08 (*p* = .37)	–	–	–	–	–	–	–
Post‐bottleneck Holarctic (1985–2017)	137	18	0.607	0.0038	0.0078	−1.34 (*p* = .06)	−11.64 (*p* < .001)	0.04 (*p* = .12)	–	–	–	–	–	–	–
Eurasia	434	40	0.787	0.0164	0.0111	1.23 (*p* = .92)	−7.83 (*p* = .07)	0.07 (*p* = .80)	91	0.509	0.633	0.201	93	6.93	–

*Note*: Mitochondrial diversity was estimated including the GenBank sequences (326 bp), while nuclear diversity was estimated using only our samples with 12 microsatellite loci. No nuclear diversity was estimated for the temporal subgroups due to insufficient sample sizes in our data. *N* – number of individuals; H – number of haplotypes; h – haplotype diversity; *π* – nucleotide diversity; *θ*_*w*_ (S) – mutation parameter theta per site calculated based on the number of segregating sites; three neutrality tests: Tajima's *D*, Fu's Fs, and Ramos‐Onsins and Rozas R_2_. *p*‐values for neutrality tests are in parentheses.

Abbreviations: A, number of alleles; AR, allelic richness; F_IS_, inbreeding coefficient; H_E_, unbiased expected heterozygosity; H_O_, observed heterozygosity; PA, number of private alleles; PAR, private allelic richness.

^a^ Western Europe was only presented in the GenBank data.

When we estimated mitochondrial diversity using only our data (*N* = 87), the sample sizes among the groups became more balanced, yet smaller (Table S9). With these, we identified 23 haplotypes (GenBank accession numbers: OR635080–OR635102; Table S10). In the entire Eurasian population, nucleotide diversity was lower than theta (*π* = 0.0067, *θ*_*w*_ = 0.0112), and the pattern remained in all groups except for Central Asia and Caucasus. The latter group also had the highest haplotype and nucleotide diversities (h = 0.752, *π* = 0.0141), while the lowest nucleotide diversity was in Northern Europe (*π* = 0.0024). Interestingly, Far East, now represented only by individuals from continental Eurasia (i.e., excluding the Japanese population), exhibited the lowest haplotype diversity (h = 0.571). Individuals carrying the Mediterranean lineage had notably higher haplotype and nucleotide diversities compared to the Holarctic lineage, but these groups had unbalanced sample sizes (*N* = 8 and 79 for Mediterranean and Holarctic, respectively).

From the microsatellite data, the H_O_ in Eurasia was 0.504, and H_E_ was 0.633. H_O_ was lower than H_E_ in all analyzed groups (Table 1). Among the four geographical groups, Far East had the highest H_O_ (0.597), and the second highest H_E_ (0.651; the highest H_E_ = 0.659 was in Central Asia and Caucasus). Inbreeding coefficient (F_IS_) was positive in all groups and varied between 0.098 (Far East) and 0.242 (Central and Eastern Europe), suggesting some inbreeding or undetected population structure (i.e., the Wahlund effect). The F_IS_ significantly deviated from zero in all groups except in Far East (Northern Europe: *V* = 49, *p* = .032; Central and Eastern Europe: *V* = 68, *p* = .021; Central Asia and Caucasus: *V* = 67, *p* = .027; Far East: *V* = 62, *p* = .077). When comparing genetic diversity among the groups, a significant difference was found only in H_O_ between Central and Eastern Europe and Far East (*W* = 36.5, *p* = .04; Table S11). Allelic richness was similar across the four groups (on average 4.23). The number of private alleles per locus (i.e., private allelic richness, PAR) in Central Asia and Caucasus was nearly twice as high as in the other groups, while Northern Europe had the lowest PAR (Table 1, Figure S3a). The two mitochondrial lineages showed similar levels of heterozygosity and inbreeding coefficients (Table 1). As these groups had a large difference in sample sizes, the results must be interpreted with caution. Nevertheless, no statistical difference was observed in the estimates of A, H_O_, H_E_, and F_IS_ between the mitochondrial lineages (Table S11). On the other hand, the Holarctic group had lower allelic and private allelic richness compared to the Mediterranean group (AR = 3.81 and 4.39, PAR = 1.08 and 1.34, respectively, corrected for sample size; Figure S3b). F_IS_ significantly deviated from zero (*V* = 74, *p* = .003) in the Holarctic group, but not in the Mediterranean group (*V* = 52, *p* = .100).

#### 3.2.2 Population structure

**FIGURE 2:**
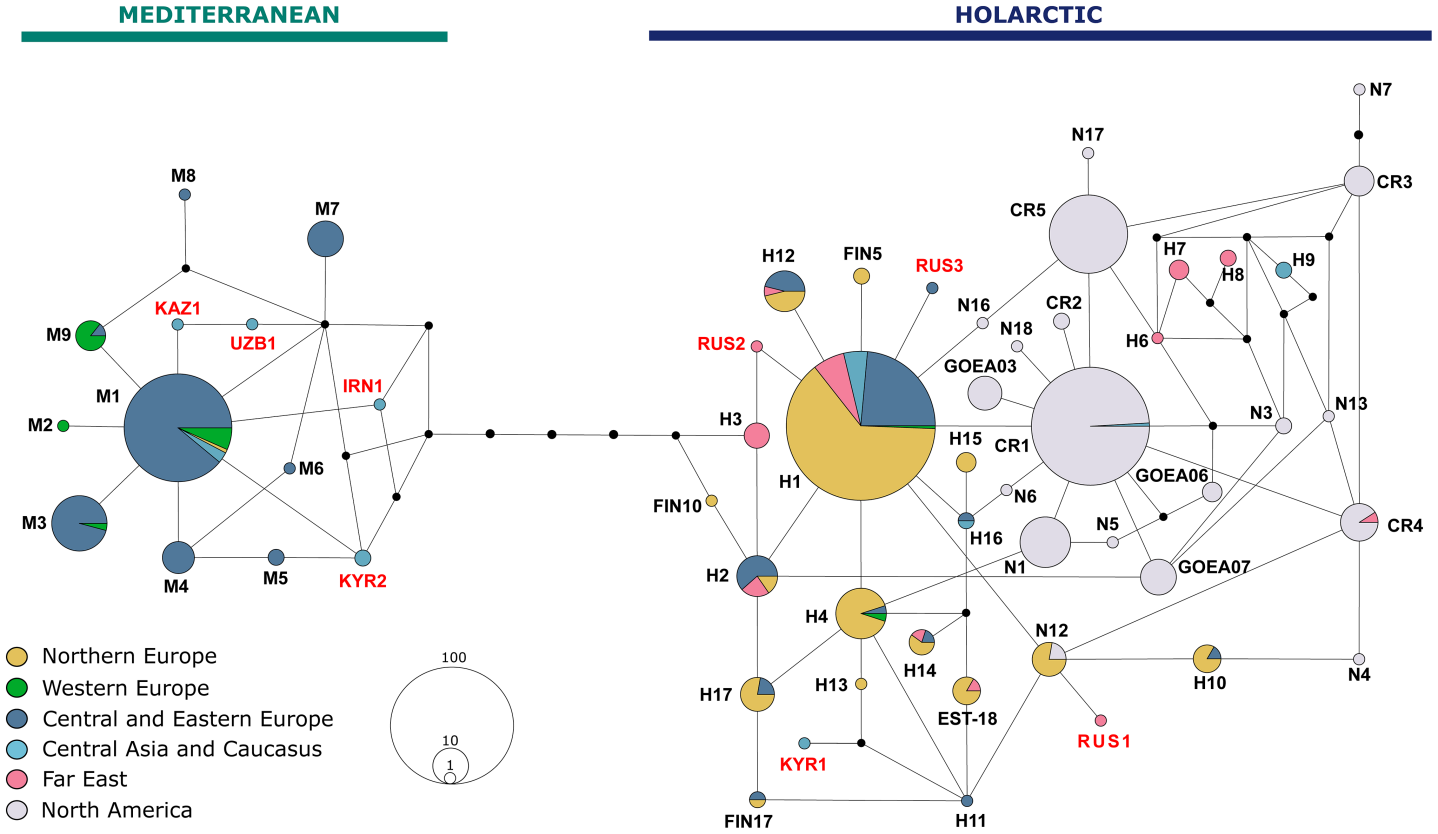
Median‐joining haplotype network of a fragment of the mtDNA CR (326 bp) from golden eagles across the Northern Hemisphere using 82 sequences from this study and 581 sequences from previous studies (Craig et al., 2016; Judkins & van den Bussche, 2017; Kylmänen et al., 2023; Nebel et al., 2015, 2019; Sonsthagen et al., 2012). The 56 haplotypes are divided into the Mediterranean and the Holarctic mitochondrial lineages. Newly found haplotypes are indicated with red font, and the names correspond to a country where the haplotype was sampled: KAZ – Kazakhstan, UZB – Uzbekistan, IRN – Iran, KYR – Kyrgyzstan, and RUS – Russia. Other haplotypes are named according to the study that has first reported the haplotype. The size of the circle corresponds to the number of individuals with a particular haplotype. Nodes indicate one mutation step.

A median‐joining haplotype network of 663 golden eagles from across the Northern Hemisphere revealed 56 haplotypes clustered into two mitochondrial lineages: Mediterranean and Holarctic (Figure 2). The Mediterranean lineage had one central haplotype M1, while the Holarctic lineage had two central haplotypes differing by one SNP: H1 in Eurasia and CR1 mainly in North America. We discovered eight new haplotypes from Russia and Central Asia (RUS1, RUS2, RUS3, KAZ1, UZB1, IRN1, KYR1, and KYR2). Haplotype CR4, previously reported only in North America, was also found in our dataset from Kamchatka. Moreover, we identified several haplotypes from the North American cluster (H6–H9) in golden eagles sampled from Far East and Central Asia and Caucasus. Thus, of the 56 haplotypes thus far detected in golden eagles, geographically three were truly Holarctic (CR1, CR4, and N12), 16 Nearctic, and 37 Palearctic. Golden eagles with Mediterranean haplotypes were found exclusively in southern Eurasia, spanning between Spain and eastern Kazakhstan, except for one individual from Northern Finland (Figure 1). On the other hand, Holarctic haplotypes were found across Eurasia (Figure 1).

From the mitochondrial data, AMOVA analyses with the GenBank sequences estimated 53.8% of genetic variation to be among the five geographical groups (*p* < .001). Furthermore, the pairwise *ɸ*_ST_ values were significant and high, pointing to existing genetic differentiation among these groups (Table 2). However, when the same analyses were performed using only our data, only 22.8% of mitochondrial variation was among the groups (*p* < .001), and the pairwise *ɸ*_ST_ ranged from −0.012 to 0.329, being significant only for Central Asia and Caucasus (Table S12). The Mediterranean and Holarctic mitochondrial lineages were highly differentiated according to pairwise *ɸ*_ST_ estimated with the GenBank sequences (*ɸ*_ST_ = 0.934, *p* < .001) and with our data only (*ɸ*_ST_ = 0.925, *p* < .001).

**TABLE 2:** Pairwise *F*_ST_ (lower diagonal) and *ɸ*_ST_ (upper diagonal) values for the geographical groups of golden eagles across Eurasia.

*F*_ST_	*ɸ*_ST_				
	Northern Europe	Central and Eastern Europe	Central Asia and Caucasus	Far East	Western Europe
Northern Europe	—	0.600 (*p* < .001)	0.548 (*p* < .001)	0.134 (*p* < .001)	0.901 (*p* < .001)
Central and Eastern Europe	0.017 (*p* = .040)	—	0.134 (*p* = .007)	0.473 (*p* < .001)	0.082 (*p* = .049)
Central Asia and Caucasus	0.038 (*p* = .004)	−0.002 (*p* = .637)	—	0.256 (*p* < .001)	0.367 (*p* < .001)
Far East	0.028 (*p* = .024)	0.003 (*p* = .386)	−0.013 (*p* = .927)	—	0.772 (*p* < .001)

*Note*: The *F*_ST_ were calculated using our data only and, thus, are presented for the four groups (Northern Europe, Central and Eastern Europe, Central Asia and Caucasus, and Far East), while the *ɸ*_ST_ were calculated with the GenBank data and included Western Europe. The *ɸ*_ST_ were calculated with Kimura 2‐parameter distance model. *p*‐values after 10,000 permutations are in parentheses.

**FIGURE 3:**
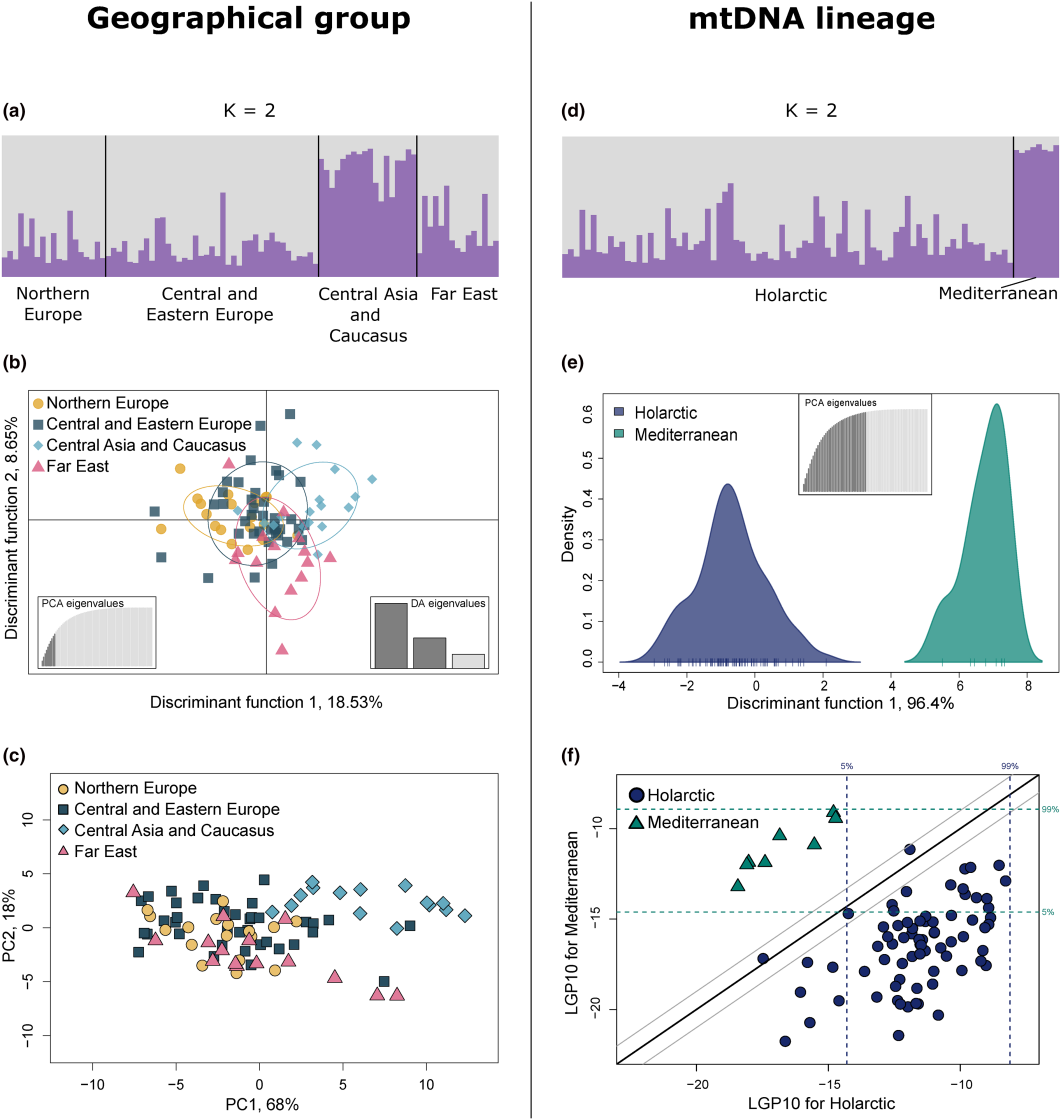
Population structure of golden eagles across Eurasia using 12 microsatellite loci. The results depict analyses of geographical groups (a–c, *N* = 91) and mitochondrial lineage groups (d–f, *N* = 87). (a) STRUCTURE results of cluster assignment of golden eagles for K = 2 using the four geographical groups as LOCPRIOR. (b) DAPC plot of the first two discriminant functions showing genetic differentiation of golden eagles from the four pre‐defined geographical groups. DAPC is based on the first ten PCs that explain 54.8% of variation. (c) PCA plot of golden eagles from the pre‐defined geographical groups. (d) STRUCTURE results of cluster assignment of golden eagles for K = 2 using a mitochondrial lineage as LOCPRIOR. (e) DAPC results of nuclear genetic differentiation of golden eagles with the Mediterranean and the Holarctic mitochondrial lineages. DAPC shows the first discriminant function and is based on the first 40 PCs that explain 96.4% of variation. (f) GenePlot of pairwise comparison of golden eagles with the Mediterranean and the Holarctic lineage haplotypes. The 5% and the 99% quantiles outline the range where genetic assignment of individuals into these groups is the most likely.

From the microsatellite data, STRUCTURE results suggested that the most likely number of genetic clusters was two when geographical locations were used as LOCPRIOR (Table S13, Figure S4), with Central Asia and Caucasus being genetically distinct from the other groups (Figure 3a). Exclusion of individuals from Central Asia and Caucasus did not result in an evident substructure among the European groups and Far East, although the best number of clusters suggested was two (Table S13, Figure S5). On the contrary to STRUCTURE results, DAPC with the four pre‐defined geographical groups did not reveal nuclear genetic structure (Figure 3b). Nevertheless, Northern Europe and Central Asia and Caucasus were somewhat separated along the Discriminant function 1. Also, PCA showed some level of differentiation of Central Asia and Caucasus (Figure 3c). The uniqueness of Central Asia and Caucasus and Northern Europe was also consistent with log genotype probability (LGP) plots of pairwise comparisons of the four geographical groups (Figure S6). Individuals with Mediterranean and Holarctic haplotypes (*N* = 87) were subdivided into two nuclear clusters by STRUCTURE when the lineage was used as LOCPRIOR (Figure 3d, Table S13, Figure S7). Nuclear differentiation of the Mediterranean and the Holarctic groups was also supported by DAPC and GenePlot results (Figure 3e,f). No population differentiation was found using the de novo STRUCTURE (Table S13, Figure S8). In the de novo DAPC, K = 2 was selected as the most likely number of clusters, but the assignment was done with only 10 PCs (Figure S9a). Besides, no spatial pattern was observed for the suggested grouping (Figure S9b). No IBD pattern was detected in Eurasia with the Mantel test (Figure S10) nor was there any spatial autocorrelation (Figure S11).

AMOVA assigned only 1.1% of nuclear variation to among the geographical groups (*p* = .07), and the pairwise microsatellite *F*_ST_ values were low and varied from −0.003 to 0.038 (Table 2). Only Northern Europe was significantly, yet weakly, differentiated (Table 2). Similarly, the nuclear *F*_ST_ between the division according to Mediterranean and Holarctic mitochondrial lineages was low and not significant (*F*_ST_ = 0.020, *p* = .120).

### 3.3 Temporal genetic variation and demographic history

**FIGURE 4:**
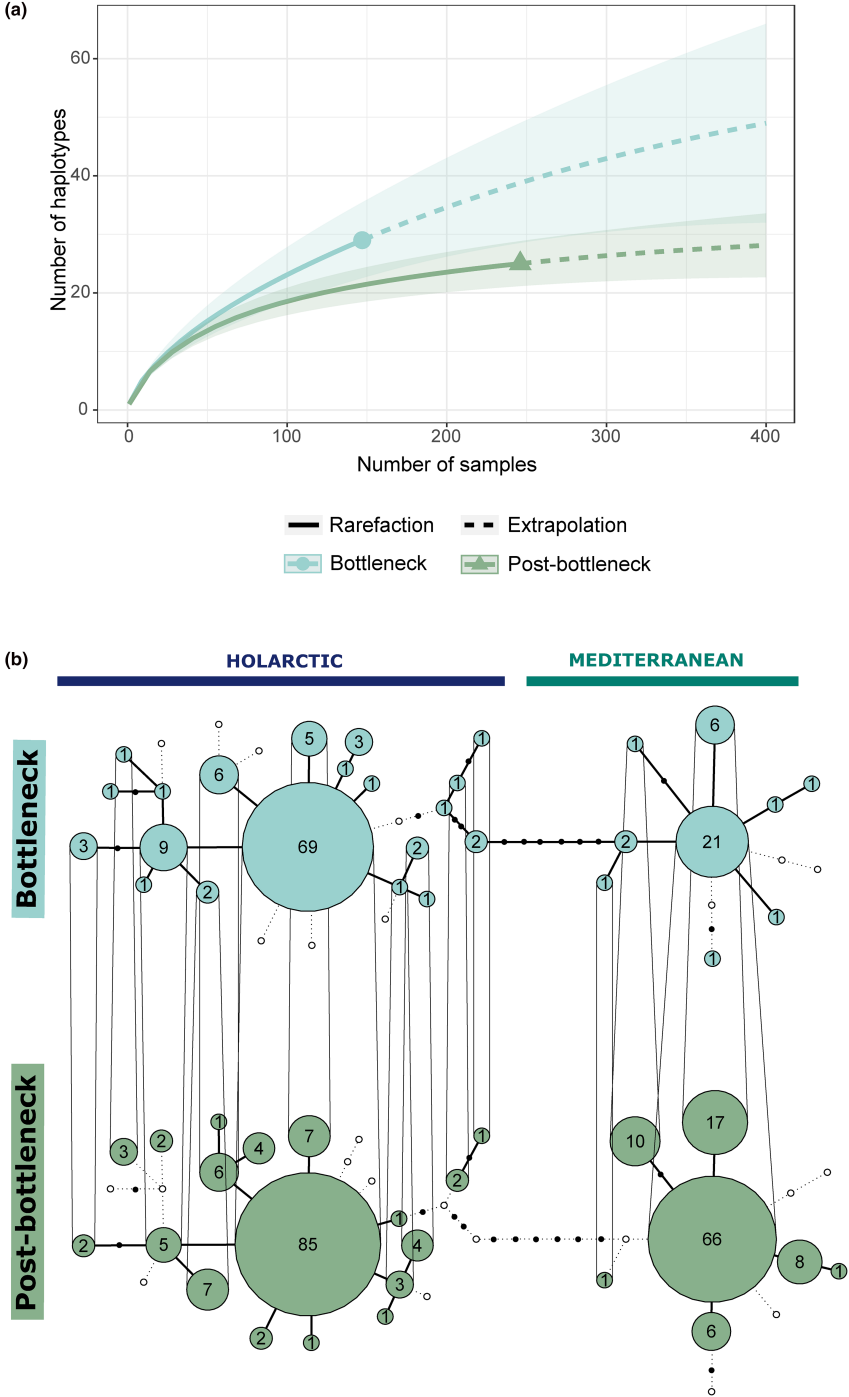
Comparison of Bottleneck (1817–1984) and Post‐bottleneck (1985–2017) temporal groups of 393 Eurasian golden eagles from a 326 bp mtDNA CR alignment. (a) Results of rarefaction‐extrapolation analyses (iNEXT) for the number of haplotypes. Circle and triangle indicate the observed number of haplotypes in both groups. (b) Temporal haplotype network, with haplotypes divided into Holarctic and Mediterranean mitochondrial lineages. The numbers in circles correspond to haplotype frequency. Haplotypes that are not found in the other group appear as small white circles. Solid lines connect extant haplotypes, and dotted lines connect the unsampled haplotypes. Nodes indicate the number of mutational steps between the haplotypes. Vertical lines connect haplotypes found in both groups.

Genetic diversity of the temporal groups is presented in Table 1. For the mitochondrial data, the Bottleneck group exhibited slightly higher haplotype and nucleotide diversities and a slightly lower theta (h = 0.754, *π* = 0.0141, *θ_W_* = 0.0121) than the Post‐bottleneck group (h = 0.800, *π* = 0.0174, *θ_W_* = 0.0101). On the contrary, rarefaction‐extrapolation analyses suggested a substantially higher number of haplotypes in the Bottleneck period compared to Post‐bottleneck (Figure 4a). When we subdivided the temporal groups into Mediterranean and Holarctic lineages, we found that mitochondrial genetic diversity was higher in the Bottleneck subgroups (Table 1). With only our data (*N* = 77), nucleotide diversity and theta were higher and haplotype diversity was lower in the Bottleneck group (h = 0.587, *π* = 0.0066, *θ*_*w*_ = 0.0103) than in the Post‐bottleneck group (h = 0.859, *π* = 0.0042, *θ*_*w*_ = 0.0074; Table S9).

A temporal haplotype network of 393 golden eagles from across Eurasia (Figure 4b) showed that the Post‐bottleneck group was missing 14 haplotypes in comparison to the Bottleneck group. Nine of these haplotypes were from the Holarctic, and five were from the Mediterranean lineage. Meanwhile, ten haplotypes not found in the Bottleneck group were discovered in the Post‐bottleneck group, of which seven were Holarctic and three Mediterranean. Central haplotypes in both Mediterranean and Holarctic lineages remained the same in both temporal groups.

**FIGURE 5:**
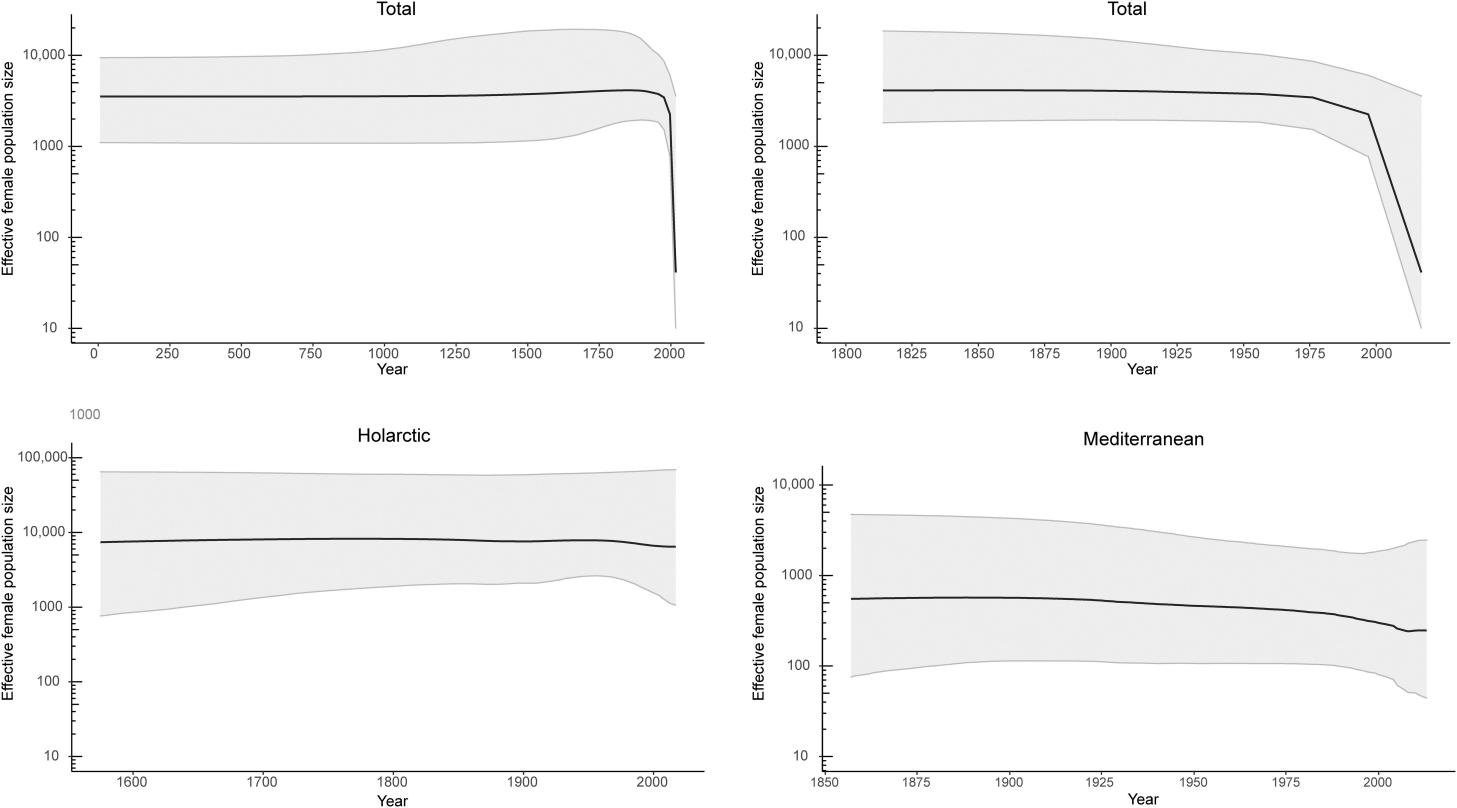
Bayesian skyline plots of effective female population sizes (N_ef_) of Eurasian golden eagles over time for the total Eurasian population (top left) zooming in to 1800–2000s (top right), and for the two mitochondrial lineages separately. Changes in N_ef_ (*y*‐axis, logarithmic scale) across time (*x*‐axis, calendar years) are presented as medians with 95% upper and lower confidence intervals. Note different scales of the axes.

The demography analyses showed signs of a previous population expansion in the Mediterranean Bottleneck subgroup as indicated by the significantly negative Tajima's D and Fu's Fs, a small significant R_2_ (Table 1), and the shape of the mismatch distributions (Figure S12). The BSP analyses revealed a decline in the effective female population size (N_ef_) in Eurasia around 1975 (Figure 5). The BSP results for the Mediterranean lineage also showed a slight declining trend during that time, while in the Holarctic lineage, the N_ef_ remained constant over the last four centuries (Figure 5). Wilcoxon's sign rank test results were not indicative of a bottleneck in any of the analyzed groups, and only the Mediterranean group had signs of population bottleneck based on the mode‐shift test (Table S14).

Among the five geographical groups, the bimodal and ragged shapes of mismatch distributions were detected for Central and Eastern Europe, Western Europe, Central Asia and Caucasus, and Far East, suggesting admixed and stable populations. Only Northern Europe showed an unimodal distribution, suggesting past population expansion (Figure S13), further supported by significantly negative Fu's Fs and R_2_, also found in Far East (Table 1).

For the microsatellite data, the observed and expected heterozygosities were at a similar level in both groups (H_O_ = 0.510 and 0.518, H_E_ = 0.628 and 0.610 for the Bottleneck and Post‐bottleneck groups, respectively). Moreover, there were no significant differences in nuclear genetic diversity (A, H_O_, H_E_, and F_IS_) between the temporal groups (Table S11), yet the inbreeding coefficient was lower in the Post‐bottleneck group (F_IS_ = 0.146 for Post‐bottleneck and 0.195 for Bottleneck). In both temporal groups, the inbreeding coefficients were positive and significantly deviated from zero (Bottleneck: *V* = 65, *p* = .005; and Post‐bottleneck: *V* = 57, *p* = .032). Allelic and private allelic richness were higher in the Bottleneck group (AR = 4.86 and 4.63, PAR = 1.08 and 0.95; Figure S3c).

While both DAPC and GenePlot showed some level of nuclear genetic differentiation between the temporal groups (Figure S14), the STRUCTURE results suggested K = 1 as the most likely number of clusters with temporal groups as LOCPRIOR (Figure S15). When we used only our data (*N* = 77), neither nuclear pairwise *F*_ST_ nor the mitochondrial *ɸ*_ST_ between the temporal groups was significant (*F*_ST_ = 0.011, *p* = .158; *ɸ*_ST_ = 0.011, *p* = .265). With the addition of the GenBank sequences, the *ɸ*_ST_ between Bottleneck and Post‐bottleneck was small yet significant (*ɸ*_ST_ = 0.075, *p* = .0001). When the temporal groups were further subdivided according to the mitochondrial lineage, the *ɸ*_ST_ values were high and significant only between the lineages, and not between the temporal periods (Table S15).

## 4 DISCUSSION

### 4.1 Genetic diversity in different parts of Eurasia

In this study, we reported both nuclear and mitochondrial genetic diversity of golden eagles for the entire Eurasia with newly sampled regions, such as Russia and Central Asia. Our analyses of nuclear microsatellites and mitochondrial sequences revealed a relatively high level of genetic variation in the Eurasian golden eagle population, being the highest in Central Asia and Caucasus, and the lowest in Northern Europe.

Mitochondrial genetic diversity in Central Asia and Caucasus (h = 0.82, *π* = 0.018) was similar to the former findings on golden eagles from mainland Asia (h = 0.79–0.93, *π* = 0.009–0.012; Nebel et al., 2015, 2023). Among the previous studies, nuclear genetic diversity was reported only for the Mongol‐Altai region, where a slightly higher observed heterozygosity (H_O_ = 0.58) but lower expected heterozygosity (H_E_ = 0.59) and allelic richness (AR = 4.07) were found compared to Central Asia and Caucasus (H_O_ = 0.51, H_E_ = 0.66, AR = 4.98). Notably, our samples from this region dated from 1898 to 1950 (*N* = 14), with four individuals of unknown years, while Nebel et al. (2023) analyzed the contemporary population. Therefore, despite the small sample size, the observed differences in nuclear genetic diversity may suggest either temporal changes or small‐scale genetic variations within mainland Asia. The high genetic diversity in Central Asia detected in our study and in the previous studies, aligns with expectations for areas near past glacial refugia (Hewitt, 2000), the central‐marginal hypothesis (CMH, Eckert et al., 2008), and the latitudinal genetic diversity gradient hypothesis (Fonseca et al., 2023; Smith et al., 2017).

On the other hand, mitochondrial diversity was the lowest, and nuclear diversity was also low in Northern Europe. While peripheral populations commonly have lower genetic diversity compared to populations at the core of the distribution (Eckert et al., 2008), the observed heterozygosity in Northern Europe (H_O_ = 0.50) was even lower than in previously studied northern European continental populations, including Finnish (H_O_ = 0.57; Kylmänen et al., 2023), Norwegian (H_O_ = 0.56; Nebel et al., 2023), and Finnish‐Estonian (H_O_ = 0.62; Nebel et al., 2023) populations, with the exception of Scotland (H_O_ = 0.46; Ogden et al., 2015). Since most of our samples from Northern Europe were from the Northwestern federal district in Russia, our results imply that this area has especially low genetic diversity compared to other northern European regions.

Central and Eastern Europe exhibited similar levels of observed heterozygosity but higher expected heterozygosity (H_O_ = 0.47, H_E_ = 0.61) compared to previously studied golden eagles in the Slovakian population (H_O_ = 0.44, H_E_ = 0.49; Bielikova et al., 2010) and in the Alps and Mediterranean region (H_O_ = 0.51, H_E_ = 0.55; Nebel et al., 2023). Additionally, we noted higher mitochondrial genetic diversity in this group compared to the Alpine and Mediterranean region (h = 0.69, *π* = 0.008; Nebel et al., 2023), regardless of whether the analyses included only our samples (h = 0.75, *π* = 0.014) or also GenBank sequences (h = 0.76, *π* = 0.016). Central and Eastern Europe group contained samples mainly originating from southern European Russia; thus, the observed high genetic diversity may point to that southern European Russia harbors a significant reservoir of genetic diversity among European golden eagles.

### 4.2 Mediterranean and Holarctic groups: genetic diversity and demographic history

By including samples from previously unexplored regions of the golden eagle distribution, we were able to better visualize the spatial distribution of the two mitochondrial lineages and compare it with the findings from nuclear markers. The Holarctic lineage was more widespread, with nearly twice as many golden eagles carrying these haplotypes (*N* = 284) compared to the Mediterranean lineage (*N* = 150). Previous studies of large raptors found that range size and historical population size were strong determinants of current genetic diversity (Väli et al., 2019). Here we discovered that the Holarctic group, occupying a larger geographical range, also had higher mitochondrial genetic diversity than the Mediterranean group. However, the latter exhibited higher allelic and private allelic richness, while other estimates of nuclear genetic diversity were comparable. The Mediterranean group also showed consistent signs of demographic expansion, which could have contributed to an increase in nuclear genetic diversity, whilst almost all demographic analyses pointed to a stable population size of the Holarctic group. The inclusion of samples from previously unstudied areas and temporal periods resulted in slight shifts in the estimated mitochondrial genetic diversity compared to the earlier reports by Nebel et al. (2015); we observed a slight reduction in haplotype and nucleotide diversities in the Holarctic group (h = 0.61 and 0.75, *π* = 0.0039 and 0.0041, in this study and in Nebel et al. (2015), respectively), and a slight increase in these parameters in the Mediterranean group (h = 0.60 and 0.58, *π* = 0.0028 and 0.0020).

Our finding of Mediterranean haplotypes in both Central and Eastern Europe and Central Asia and Caucasus suggests that the Mediterranean lineage is spread more eastwards than thought before, where it now coexists with the Holarctic lineage, resulting in high genetic diversity in this region. The Mediterranean lineage likely survived in a glacial refugium around the Mediterranean region, as previously suggested by Nebel et al. (2015), but the location of a refugium for the Holarctic lineage remains uncertain; perhaps it was somewhere in central‐eastern Asia. The Mongolian Plateau and the Altai‐Saiyan Mountains have been suggested as glacial refugia for several plant and mammal species (Hais et al., 2015; Lv et al., 2016; McLean et al., 2018), making them plausible candidates also for the golden eagle, especially in the light of the recently discovered golden eagle's genetic diversity hotspot in the Mongol‐Altai region (Nebel et al., 2023). Another possibility is that rather than being restricted to a single refugium, golden eagles of the Holarctic lineage had a wide and continuous distribution south and east from the continental ice sheets, as has been shown for example in some mammals, including the hare (*Lepus timidus*) and the caribou (*Rangifer tarandu*s; Wang et al., 2021).

### 4.3 The north–south genetic gradient in Eurasian golden eagles

We discovered both a north–south genetic gradient and genetic differentiation among the geographical groups in Eurasian golden eagles with both mitochondrial and microsatellite analyses. The divergence suggested by mitochondrial markers was consistently larger than that estimated by microsatellites, which was expected based on faster lineage sorting of mitochondrial markers due to their smaller effective size compared to microsatellites. While the two mitochondrial lineages were identified and comprehensively discussed by Nebel et al. (2015), no evidence of their division was associated with nuclear markers. In addition, we highlighted the genetic uniqueness of the Central Asia and Caucasus and the Northern Europe groups in Eurasian golden eagles.

The latitudinal genetic gradient can originate due to several factors, including climatic and environmental instability in the north (Eckert et al., 2008; Smith et al., 2017), differences in migratory flyways (Monti et al., 2018), postglacial colonization history (Thörn et al., 2021), and a combined influence of the Quaternary climatic changes (Fonseca et al., 2023). For example, a large‐scale study of ospreys (*Pandion haliaetus*), identified two genetic clusters in Eurasia: Mediterranean and Eurasian, which were attributed to different migratory flyways (Monti et al., 2018). Interestingly, the genetic clustering of ospreys was geographically similar to the genetic clustering observed in Eurasian golden eagles. However, the migratory flyway theory is not applicable to golden eagles because Eurasian golden eagles are generally non‐migratory (Watson, 2010). On the other hand, the genetic differentiation between the north and the south as a result of post‐glacial colonization history is supported by our findings of genetic diversity and demographic history (see above). Furthermore, golden eagles occupy a variety of habitats, implying diverse dietary and nesting adaptations. Their distribution in southern Eurasia covers Mediterranean‐rich habitats, mountains, and steppes, while in northern Eurasia, they are predominantly found in mixed forests and taiga (Watson, 2010). This distinct ecological variation may be a potential underlying reason for the observed genetic differentiation.

However, although this gradient resulting from mixing of two divergent lineages seems to exist, we did not find evidence for isolation by distance (IBD) among the Eurasian golden eagles. Lack of an IBD pattern may be explained by the high dispersal capacity of golden eagles, especially of adolescent birds, documented in multiple studies (Nygård et al., 2016; Poessel et al., 2022). High dispersal potential leading to high gene flow was also mentioned as a reason for a lack of the IBD pattern in British golden eagles (Bourke et al., 2010) and in some large‐scale studies of other philopatric raptors, such as the Eurasian kestrel (*Falco tinnunculus*; Alcaide et al., 2009) and the greater spotted eagle (*Clanga clanga*; Väli et al., 2019).

### 4.4 Temporal genetic variation and demographic history

The population bottleneck of the 19th and 20th centuries has left genetic signs in Palearctic golden eagles. First, we noticed a decrease in the number of haplotypes. While it is possible that the absence of 14 haplotypes in the post‐bottleneck period compared to the bottleneck period was due to incomplete sampling, the rarefaction‐extrapolation analyses indicated that the Bottleneck group had a significantly higher number of haplotypes compared to the Post‐bottleneck group. Second, we observed a decrease in allelic and private allelic richness; a signal of a population bottleneck, as rare alleles are lost at a faster rate than heterozygosity is decreased (Allendorf, 1986). Third, the Bayesian Skyline Plot (BSP) analyses showed a reduction in the effective female population size (N_ef_) in the Eurasian population starting around 1975, aligning with known population declines in many populations across Eurasia (Bielikova et al., 2010; Nebel et al., 2015; Ollila, 2018; Starikov, 2020). Finally, when comparing temporal variation between the Mediterranean and Holarctic lineages, we found that in both lineages, genetic diversity was higher in the Bottleneck groups. This highlights the potential consequences of the recent bottleneck, which led to a decrease in the genetic diversity of the post‐bottleneck population.

However, there are some potential sources of bias in genetic diversity estimates. First, the temporal groups differ in the number of samples from different regions, especially for the high‐diversity area of Central Asia and Caucasus (*N* = 17 in the Bottleneck group, and *N* = 1 in the Post‐bottleneck group). Second, reproductive state of the sampled individuals may have an effect (especially, age and season) because golden eagles perform extensive dispersal during their first 4–5 years and there is seasonal variation in movement of both preadult and adult birds (Sur et al., 2020; Watson, 2010). Consequently, birds sampled from the area do not necessarily represent the breeding population. Although we knew the age group of most sampled individuals in the main dataset, we did not know the month of sampling. Furthermore, for the GenBank data, information on age and sampling season was unavailable.

Despite the population bottleneck, golden eagles have nevertheless retained relatively high levels of genetic diversity. Factors such as long generation time, admixed origin of populations, and large distribution range contribute to high genetic diversity (Avise, 2000; Hailer et al., 2006; Väli et al., 2019). Therefore, as golden eagles are long‐lived (Watson, 2010), occupy vast geographical areas (BirdLife International, Handbook of the Birds of the World, 2022), and likely originate from several glacial refugia (Nebel et al., 2015; see above), these factors have undoubtedly played an important role in maintaining their genetic diversity. Similarly, high genetic diversity has been observed in other Eurasian raptors, such as the white‐tailed eagle (Hailer et al., 2007), the cinerous vulture (*Aegypius monachus*, Poulakakis et al., 2008), the crowned solitary eagle (*Buteogallus coronatus*; Canal et al., 2017), and the greater spotted eagle (Väli et al., 2019).

While the long generation time may have buffered the effects of the 19th‐ and 20th‐century bottleneck, it is also possible that the recent population decline was not severe enough to cause significant reductions in golden eagle's genetic diversity (Bourke et al., 2010). Noteworthy, the bottleneck of the 19th and 20th centuries might have occurred at various time points in different parts of Eurasia, distorting the detection of temporal genetic diversity changes on a large scale. Unfortunately, no studies on long‐term population trends in golden eagles have been conducted in Russia or elsewhere in continental Asia. For example, the only available records in central Yakutia state that the species was commonly nesting until the mid‐1950s but became rare and disappeared from some areas in 1970s–1980s, and only during the last 15–20 years the population has started to grow (Isaev et al., 2021). Similarly, no golden eagle nests were found in Dauriya (east of the Lake Baikal, Zabaikalskiy krai) for the period from 1950s until 1990s (Karyakin & Nikolenko, 2012). On the other hand, nesting in the upper parts of the Don River basin (European Russia) was questioned already in the beginning of the 20th century, but the encounters became more frequent since the mid‐1960s (Semago, 2006). Similarly, in Kazakh uplands (Kazakhstan), golden eagles were only reported since 1960s (Starikov, 2020).

When interpreting the population history of golden eagles, it is essential also to consider the significant impact of major glaciations on the distribution and dynamics of species (Hewitt, 2004). The glacial periods have had a huge impact on species' ranges through dispersal, contractions, and even extinctions (Hewitt, 2000). In our study, we detected signals of population expansion of golden eagles in Northern Europe, a region that was long covered by the Scandinavian Ice Sheet. During the last glacial period, the Scandinavian Ice Sheet was the largest component of the Eurasian ice sheet complex, and it covered Fennoscandia and North‐Western Russia repeatedly (Hughes et al., 2016). Upon the end of the Last Glacial Maximum (26.5–19 ka; Clark et al., 2009), species began to re‐colonize new regions as the ice retreated (Behzadi et al., 2022; Ersmark et al., 2019). Although the precise routes of recolonization of Northern Europe remain uncertain, one possible direction could have been from the east of Eurasia, due to the prevalence of Holarctic haplotypes over the Mediterranean ones in this region.

## 5 CONCLUSIONS

In this study, we performed a combination of microsatellite and mtDNA analyses using samples from previously unexamined regions of the golden eagle's distribution to enhance our understanding of the phylogeography of this species across Eurasia. Our findings revealed genetic differentiation between the Mediterranean and the Holarctic lineages and a divergence of Northern Europe and Central Asia and Caucasus from the other studied regions, thereby highlighting a latitudinal genetic gradient between southern and northern Eurasian golden eagles. Upon comparing these genetic clusters, we found that Central Asia was a hotspot of genetic diversity, while Northern Europe showed an opposite trend.

Although we lack definitive conclusions to fully explain the latitudinal gradient of golden eagles, the presence of two genetically distinct lineages with differing demographic histories and geographical distributions provides evidence for the existence of two evolutionarily significant units (ESU; sensu Crandall et al., 2000). Incorporating ESUs into conservation and management practices would help to preserve the adaptive diversity of this remarkable species (Crandall et al., 2000). Finally, conservation of golden eagles is not only a matter of national concern but requires consideration at a larger scale to preserve this distinct genetic variation.

## AUTHOR CONTRIBUTIONS

**Ekaterina Karabanina:** Conceptualization (equal); data curation (lead); formal analysis (lead); funding acquisition (supporting); investigation (lead); methodology (equal); visualization (lead); writing – original draft (lead); writing – review and editing (equal). **Gerhardus M. J. Lansink:** Conceptualization (equal); formal analysis (supporting); investigation (supporting); methodology (equal); supervision (equal); writing – review and editing (equal). **Suvi Ponnikas:** Conceptualization (equal); methodology (equal); supervision (equal); writing – review and editing (equal). **Laura Kvist:** Conceptualization (equal); data curation (supporting); funding acquisition (lead); methodology (equal); project administration (lead); supervision (equal); writing – review and editing (equal).

## FUNDING INFORMATION

This research was funded by the Kvantum Institute of the University of Oulu. E.K. has also received funding from the Societas pro Fauna et Flora Fennica and the Oulun yliopiston tukisäätiö.

## CONFLICT OF INTEREST STATEMENT

The authors declare no conflict of interest.

## Supporting information


Appendix S1.

Data S1.

## Data Availability

DNA sequences are available from GenBank under accession numbers OR635080–OR635102 and microsatellite genotyping data in Supporting Information.
