# Supplementary material for: A renewed glance at the Palearctic golden eagle: Genetic variation in space and time
Source: Ecol Evol. 2024 Mar 10;14(3):e11109. doi: 10.1002/ece3.11109 (PMC10925523; doi:10.1002/ece3.11109)
Supplement: Supplementary file 1 — Appendix S1. [file ECE3-14-e11109-s001.docx]

**Supporting information**

**A renewed glance at the Palearctic golden eagle: genetic variation in space and time**

Ekaterina Karabanina^*^, Gerhardus M.J. Lansink, Suvi Ponnikas, Laura Kvist

Department of Ecology & Genetics, P.O. Box 3000, 90014 University of Oulu, Finland

^*^[ekaterina.karabanina@oulu.fi](mailto:ekaterina.karabanina@oulu.fi)

**Contents**

**Figure S1.** Map with locations of golden eagle samples used in this study.

**Table S1.** Description of 91 golden eagle samples used in this study.

**Table S2.** Description of the 12 microsatellite loci primers used for genotyping 91 golden eagles.

**Table S3.** Accession numbers to GenBank sequences that were added to this study.

**Table S4.** Summary of the number of samples used for different mtDNA analyses with and without the GenBank data.

**Table S5.** Summary of how samples from this study and GenBank were assigned to five geographical groups.

**Table S6**. Calculation of golden eagle’s replacement (generation) time.

**Table S7.** Summary table of the Tracer output for the BEAST analyses of Eurasian golden eagles.

**Table S8.** Summary statistics on 12 microsatellite loci used in genotyping 91 golden eagles.

**Figure S2.** Results of rarefaction-extrapolation analyses (iNEXT) for the number of haplotypes found in different groups of Eurasian golden eagles.

**Table S9.** Mitochondrial genetic diversity of 87 Eurasian golden eagles using only our data (CR, 390 bp).

**Table S10.** 23 golden eagle haplotypes found in this study.

**Table S11.** Results of the Mann-Whitney U test (Wilcoxon rank sum test) and the Kruskal-Wallis tests for comparing genetic diversity estimates among groups of 91 Eurasian golden eagles.

**Figure S3.** The mean private allelic richness (number of private alleles per locus) as a function of standardized sample size for the 91 Eurasian golden eagles divided into three groups.

**Table S12.** Pairwise ɸ_ST_ values for the four geographical groups (Northern Europe, Central and Eastern Europe, Central Asia and Caucasus, and Far East) of Eurasian golden eagles using only our data (N = 87).

**Table S13.** Summary of the STRUCTURE runs of cluster assignment of golden eagles using 12 microsatellite loci.

**Figure S4.** STRUCTURE results of cluster assignment of 91 golden eagles from the four geographical groups based on 12 microsatellite loci for K = 1 to 5.

**Figure S5.** STRUCTURE results of cluster assignment of golden eagles excluding individuals from Central Asia and Caucasus.

**Figure S6.** Geneplot results of pairwise comparisons of the four geographical groups of golden eagles with minimum of eight loci genotyped.

**Figure S7.** STRUCTURE results of cluster assignment of 87 golden eagles grouped according to the mitochondrial lineage (Holarctic and Mediterranean) using 12 microsatellite loci for K = 1 to 5.

**Figure S8.** STRUCTURE results of cluster assignment of 91 golden eagles without LOCPRIOR information based on 12 microsatellite loci for K = 1 to 5.

**Figure S9.** DAPC results of the *de novo* grouping of 91 golden eagles into two clusters (K = 2) according to the lowest BIC value.

**Figure S10.** Mantel test for 91 Eurasian golden eagles using 12 microsatellites.

**Figure S11.** Correlogram of spatial autocorrelation analysis for 91 Eurasian golden eagles using 12 microsatellites.

**Figure S12.** Mismatch distributions graphs for 393 Eurasian golden eagles from Mediterranean and Holarctic lineages, further split into temporal groups: Bottleneck and Post-bottleneck.

**Table S14.** The Bottleneck program analyses results.

**Figure S13.** Mismatch distributions graphs for 434 Eurasian golden eagles from the five geographical groups: Northern Europe, Central and Eastern Europe, Central Asia and Caucasus, Far East, and Western Europe.

**Figure S14.** DAPC and GenePlot results for Eurasian golden eagles from Bottleneck and Post-bottleneck groups.

**Figure S15**. STRUCTURE results of cluster assignment of 80 golden eagles grouped according to the temporal group (Bottleneck and Post-bottleneck) using 12 microsatellite loci for K = 1 to 5.

**Table S15.** Pairwise ɸ_ST_ values for temporal groups of Eurasian golden eagles, subdivided according to the mitochondrial lineage.


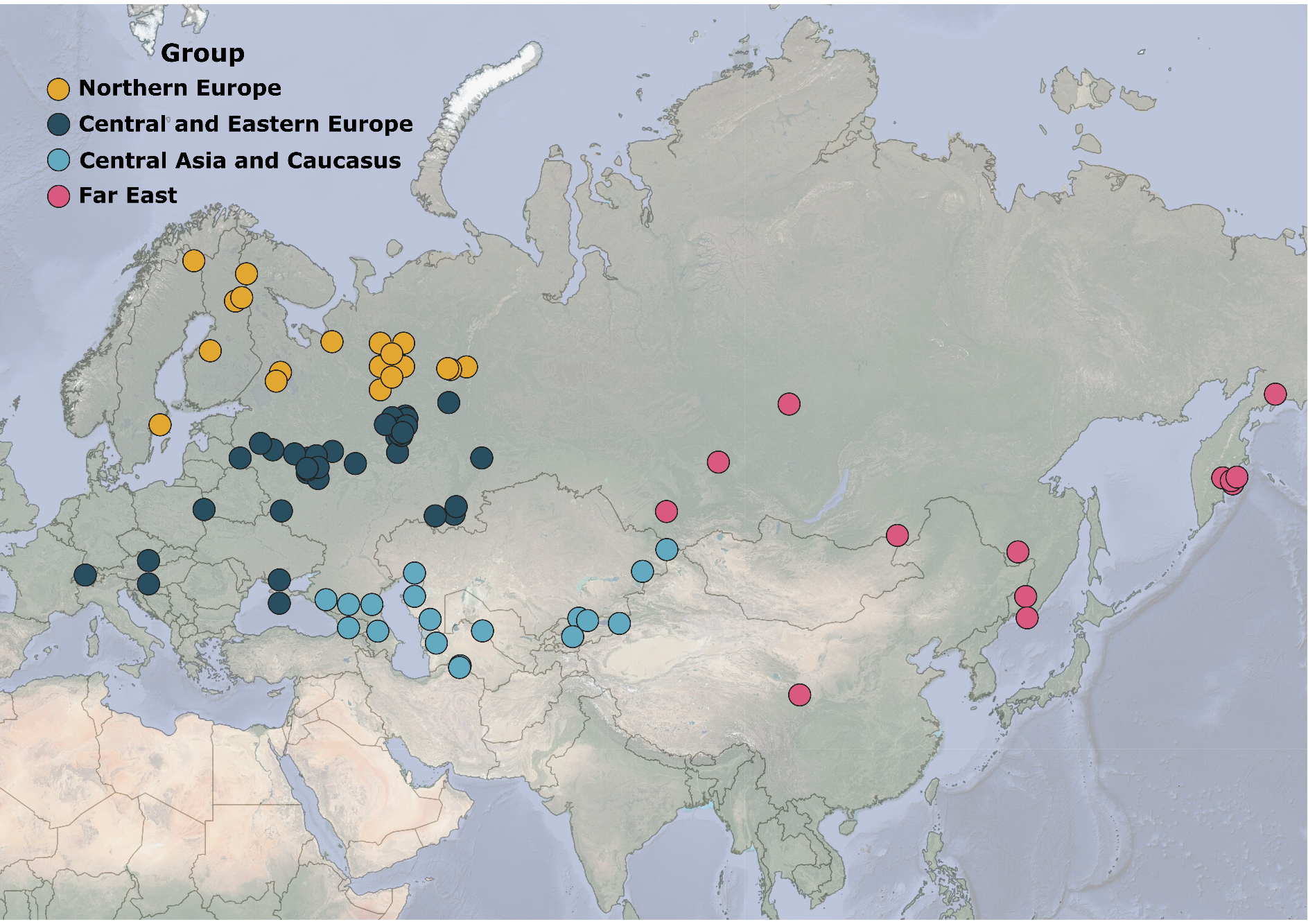


**Figure S1.** Map with locations of golden eagle samples used in this study. Geographical groups are color-marked: Northern Europe – yellow, Central and Eastern Europe – dark teal, Central Asia and Caucasus – light blue, and Far East – pink. The map was created in QGIS 3.10 (QGIS Development Team, 2022).

**Table S1.** Description of 91 golden eagle samples used in this study (i.e., the main dataset). The museum feather was plucked from a museum specimen. Feather samples from captivity were collected as shed feathers of captive individuals (whose origins were known). Unknown year is marked as NA. Geographical groups are based on locations of individuals and are given as used in the analyses. Subspecies are named according to geographical locations. Haplotype codes are given as in the haplotype network (main text Figure 1), or as submitted to GenBank. Four individuals were not analyzed for mtDNA due to short sequence lengths, therefore, mtDNA lineage and haplotype are not given for these individuals. Asterisks indicate samples from the former study by Kylmänen *et al.* (2023).

| **ID** | **Material** | **Source** | **Year** | **Country** | **Geographical group** | **Subspecies** | **mtDNA lineage** | **Haplotype (as in haplotype network)** | **Haplotype (as submitted to GenBank)** |
| --- | --- | --- | --- | --- | --- | --- | --- | --- | --- |
| AC44* | feather | nest | 2007 | Finland | Northern Europe | *A.c.chrysaetos* | Holarctic | H4 | FIN2 |
| AC835* | feather | nest | 2014 | Finland | Northern Europe | *A.c.chrysaetos* | Holarctic | H1 | FIN11 |
| AC1034* | feather | nest | 2015 | Finland | Northern Europe | *A.c.chrysaetos* | Holarctic | H1 | FIN1 |
| AC1055* | feather | nest | 2015 | Finland | Northern Europe | *A.c.chrysaetos* | Holarctic | H1 | FIN1 |
| AC1101* | feather | nest | 2014 | Finland | Northern Europe | *A.c.chrysaetos* | Holarctic | H1 | FIN1 |
| AC1251 | feather | museum | 1972 | Russia | Northern Europe | *A.c.chrysaetos* | Holarctic | H1 | RUS17 |
| AC1252 | feather | captivity | 1996 | Russia | Northern Europe | *A.c.chrysaetos* | Holarctic | FIN5 | RUS4 |
| AC1254B | feather | captivity | 2014 | Russia | Central and Eastern Europe | *A.c.chrysaetos* | Holarctic | H1 | RUS17 |
| AC1254C | feather | captivity | 2017 | Russia | Central and Eastern Europe | *A.c.chrysaetos* | Holarctic | H1 | RUS6 |
| AC1255 | skin | museum | 1926 | Russia | Central and Eastern Europe | *A.c.chrysaetos* | Holarctic | H1 | RUS17 |
| AC1256 | skin | museum | 1927 | Russia | Central and Eastern Europe | *A.c.chrysaetos* | Holarctic | H1 | RUS17 |
| AC1257 | skin | museum | 1927 | Russia | Central and Eastern Europe | *A.c.chrysaetos* | Holarctic | H1 | RUS17 |
| AC1258 | skin | museum | 1929 | Russia | Central and Eastern Europe | *A.c.chrysaetos* | Holarctic | H1 | RUS16 |
| AC1259 | skin | museum | 1932 | Russia | Northern Europe | *A.c.chrysaetos* | Holarctic | H1 | RUS17 |
| AC1260 | skin | museum | 1932 | Russia | Northern Europe | *A.c.chrysaetos* | Holarctic | H12 | RUS5 |
| AC1261 | skin | museum | 1932 | Russia | Northern Europe | *A.c.chrysaetos* | Holarctic | N12 | RUS7 |
| AC1262 | skin | museum | 1932 | Russia | Northern Europe | *A.c.chrysaetos* | Holarctic | H1 | RUS17 |
| AC1263 | skin | museum | 1932 | Russia | Northern Europe | *A.c.chrysaetos* | Holarctic | H1 | RUS17 |
| AC1264 | skin | museum | 1932 | Russia | Northern Europe | *A.c.chrysaetos* | Holarctic | H1 | RUS17 |
| AC1265 | skin | museum | 1932 | Russia | Northern Europe | *A.c.chrysaetos* | Holarctic | H14 | RUS14 |
| AC1266 | skin | museum | 1912 | Sweden | Northern Europe | *A.c.chrysaetos* | Holarctic | H1 | RUS17 |
| AC1267 | skin | museum | 1912 | Russia | Central Asia and Caucasus | *A.c.homeyeri* | Mediterranean | KYR2 | KYR2 |
| AC1268 | skin | museum | 1913 | Russia | Central Asia and Caucasus | *A.c.homeyeri* | Holarctic | H1 | RUS17 |
| AC1269 | skin | museum | 1914 | Russia | Central Asia and Caucasus | *A.c.homeyeri* | Holarctic | H1 | RUS17 |
| AC1270 | skin | museum | NA | Switzerland | Central and Eastern Europe | *A.c.chrysaetos* | Holarctic | H1 | RUS17 |
| AC1271 | skin | museum | 1931 | Uzbekistan | Central Asia and Caucasus | *A.c.daphanea* | Mediterranean | UZB1 | UZB1 |
| AC1272 | skin | museum | 1911 | Russia | Central and Eastern Europe | *A.c.chrysaetos* | Holarctic | H1 | RUS17 |
| AC1273 | skin | museum | 1911 | Kyrgyzstan | Central Asia and Caucasus | *A.c.daphanea* | Holarctic | H1 | RUS17 |
| AC1274 | skin | museum | NA | Russia | Far East | *A.c.kamtschatica* | Holarctic | H1 | RUS17 |
| AC1275 | skin | museum | NA | Russia | Far East | *A.c.kamtschatica* | Holarctic | H1 | RUS17 |
| AC1276 | skin | museum | 1934 | Kyrgyzstan | Central Asia and Caucasus | *A.c.daphanea* | Holarctic | KYR1 | KYR1 |
| AC1277 | skin | museum | 1913 | Azerbaijan | Central Asia and Caucasus | *A.c.homeyeri* | Holarctic | H1 | RUS17 |
| AC1278 | skin | museum | NA | Russia | Central and Eastern Europe | *A.c.daphanea* | - | - | - |
| AC1279 | skin | museum | 1934 | Russia | Central and Eastern Europe | *A.c.chrysaetos* | - | - | - |
| AC1280 | skin | museum | NA | Austria | Central and Eastern Europe | *A.c.chrysaetos* | Mediterranean | M1 | KAZ2 |
| AC1281 | skin | museum | NA | Austria | Central and Eastern Europe | *A.c.chrysaetos* | Holarctic | H1 | RUS17 |
| AC1282 | skin | museum | 1934 | Russia | Far East | *A.c.kamtschatica* | Holarctic | H1 | RUS17 |
| AC1283 | skin | museum | NA | Belarus | Central and Eastern Europe | *A.c.chrysaetos* | Holarctic | H4 | RUS16 |
| AC1284 | skin | museum | 1935 | Russia | Central and Eastern Europe | *A.c.chrysaetos* | Holarctic | H1 | RUS17 |
| AC1285 | skin | museum | 1921 | Ukraine | Central and Eastern Europe | *A.c.chrysaetos* | Holarctic | H1 | RUS17 |
| AC1286 | skin | museum | 1922 | Ukraine | Central and Eastern Europe | *A.c.chrysaetos* | Holarctic | H1 | RUS17 |
| AC1287 | skin | museum | 1937 | Russia | Northern Europe | *A.c.chrysaetos* | Holarctic | H1 | RUS17 |
| AC1288 | skin | museum | 1937 | Russia | Northern Europe | *A.c.chrysaetos* | Holarctic | H4 | RUS16 |
| AC1289 | skin | museum | 1932 | Russia | Central and Eastern Europe | *A.c.chrysaetos* | Holarctic | H1 | RUS17 |
| AC1290 | skin | museum | 1937 | Russia | Central and Eastern Europe | *A.c.chrysaetos* | Holarctic | H1 | RUS17 |
| AC1291 | skin | museum | 1938 | Russia | Central and Eastern Europe | *A.c.chrysaetos* | Holarctic | H1 | RUS17 |
| AC1292 | skin | museum | 1938 | Russia | Central and Eastern Europe | *A.c.chrysaetos* | Holarctic | H1 | RUS17 |
| AC1293 | skin | museum | 1939 | Russia | Northern Europe | *A.c.chrysaetos* | Holarctic | H1 | RUS17 |
| AC1294 | skin | museum | 1941 | Russia | Central and Eastern Europe | *A.c.chrysaetos* | Holarctic | H1 | RUS17 |
| AC1295 | skin | museum | 1931 | Kazakhstan | Central Asia and Caucasus | *A.c.daphanea* | Holarctic | H1 | RUS17 |
| AC1296 | skin | museum | 1929 | Russia | Far East | *A.c.kamtschatica* | Holarctic | H1 | RUS17 |
| AC1297 | skin | museum | 1942 | Turkmenistan | Central Asia and Caucasus | *A.c.daphanea* | Mediterranean | M1 | KAZ2 |
| AC1298 | skin | museum | 1937 | Russia | Central and Eastern Europe | *A.c.chrysaetos* | - | - | - |
| AC1299 | skin | museum | 1930 | Russia | Central and Eastern Europe | *A.c.chrysaetos* | - | - | - |
| AC1300 | skin | museum | 1928 | Kyrgyzstan | Central Asia and Caucasus | *A.c.daphanea* | Mediterranean | KYR2 | KYR2 |
| AC1301 | skin | museum | NA | Kazakhstan | Central Asia and Caucasus | *A.c.daphanea* | Holarctic | H1 | RUS17 |
| AC1302 | skin | museum | NA | Kazakhstan | Central Asia and Caucasus | *A.c.daphanea* | Holarctic | H1 | RUS17 |
| AC1303 | skin | museum | NA | Kazakhstan | Central Asia and Caucasus | *A.c.daphanea* | Mediterranean | M1 | KAZ2 |
| AC1304 | skin | museum | 1946 | Russia | Far East | *A.c.kamtschatica* | Holarctic | RUS1 | RUS1 |
| AC1305 | skin | museum | 1946 | Russia | Far East | *A.c.kamtschatica* | Holarctic | EST-18 | RUS8 |
| AC1306 | skin | museum | 1946 | Russia | Central and Eastern Europe | *A.c.chrysaetos* | Holarctic | H2 | RUS15 |
| AC1307 | skin | museum | NA | Russia | Central Asia and Caucasus | *A.c.homeyeri* | Holarctic | H16 | RUS9 |
| AC1308 | skin | museum | 1922 | Russia | Central and Eastern Europe | *A.c.chrysaetos* | Holarctic | H1 | RUS17 |
| AC1309 | skin | museum | 1947 | Turkmenistan | Central Asia and Caucasus | *A.c.daphanea* | Holarctic | H1 | RUS17 |
| AC1310 | skin | museum | 1950 | Russia | Far East | *A.c.kamtschatica* | Holarctic | H1 | RUS17 |
| AC1311 | skin | museum | 1949 | Russia | Far East | *A.c.kamtschatica* | Holarctic | H8 | RUS10 |
| AC1312 | skin | museum | 1950 | Russia | Northern Europe | *A.c.chrysaetos* | Holarctic | H1 | RUS17 |
| AC1313 | skin | museum | 1950 | Kazakhstan | Central Asia and Caucasus | *A.c.daphanea* | Mediterranean | KAZ1 | KAZ1 |
| AC1314 | skin | museum | 1951 | Russia | Far East | *A.c.kamtschatica* | Holarctic | H1 | RUS17 |
| AC1315 | skin | museum | 1929 | Russia | Central and Eastern Europe | *A.c.chrysaetos* | Holarctic | H4 | RUS16 |
| AC1316 | skin | museum | 1925 | Iran | Central Asia and Caucasus | *A.c.homeyeri* | Mediterranean | IRN1 | IRN1 |
| AC1317 | skin | museum | 1954 | Russia | Central and Eastern Europe | *A.c.chrysaetos* | Holarctic | H1 | RUS17 |
| AC1318 | skin | museum | NA | Kyrgyzstan | Central Asia and Caucasus | *A.c.daphanea* | Holarctic | H1 | RUS17 |
| AC1319 | skin | museum | 1958 | Russia | Far East | *A.c.kamtschatica* | Holarctic | H1 | RUS17 |
| AC1320 | skin | museum | 1960 | Russia | Central and Eastern Europe | *A.c.chrysaetos* | Holarctic | H1 | RUS17 |
| AC1321 | skin | museum | NA | China | Far East | *A.c.daphanea* | Holarctic | H1 | RUS17 |
| AC1322 | skin | museum | 1960 | Russia | Far East | *A.c.kamtschatica* | Holarctic | H1 | RUS17 |
| AC1323 | skin | museum | 1975 | Russia | Far East | *A.c.kamtschatica* | Holarctic | RUS2 | RUS2 |
| AC1324 | skin | museum | 1971 | Russia | Far East | *A.c.kamtschatica* | Holarctic | H1 | RUS17 |
| AC1325 | skin | museum | 1989 | Russia | Central and Eastern Europe | *A.c.chrysaetos* | Holarctic | RUS3 | RUS3 |
| AC1326 | skin | museum | 2003 | Russia | Central and Eastern Europe | *A.c.chrysaetos* | Holarctic | H1 | RUS17 |
| AC1327 | skin | museum | 2011 | Russia | Far East | *A.c.kamtschatica* | Holarctic | CR4 | RUS11 |
| AC1328 | skin | museum | 2014 | Russia | Central and Eastern Europe | *A.c.chrysaetos* | Holarctic | H4 | RUS16 |
| AC1329 | skin | museum | 1927 | Russia | Central and Eastern Europe | *A.c.chrysaetos* | Holarctic | H4 | RUS16 |
| AC1330 | skin | museum | 1956 | Russia | Central and Eastern Europe | *A.c.chrysaetos* | Holarctic | H14 | RUS14 |
| AC1331 | skin | museum | 1948 | Russia | Central and Eastern Europe | *A.c.chrysaetos* | Holarctic | H1 | RUS17 |
| AC1332 | skin | museum | 1938 | Russia | Central and Eastern Europe | *A.c.chrysaetos* | Holarctic | H2 | RUS15 |
| AC1333 | skin | museum | 1953 | Russia | Central and Eastern Europe | *A.c.chrysaetos* | Holarctic | H1 | RUS17 |
| AC1334 | skin | museum | 1960 | Russia | Central and Eastern Europe | *A.c.chrysaetos* | Holarctic | H1 | RUS12 |
| AC1335 | skin | museum | 2000 | Russia | Central and Eastern Europe | *A.c.chrysaetos* | Holarctic | FIN17 | RUS13 |
| AC1336 | skin | museum | 1928 | Russia | Central and Eastern Europe | *A.c.chrysaetos* | Holarctic | H4 | RUS16 |

**Table S2.** Description of the 12 microsatellite loci primers for genotyping golden eagles. The range of the possible fragment sizes is given based on this study. “Original species” indicates the species for which the primers were originally developed.

| **Locus** | **Primer sequences 5′−3′** | **Repeat motif** | **Size, bp** | **Original species** | **Reference** | **Primer concentration, μM** | **Dye** |
| --- | --- | --- | --- | --- | --- | --- | --- |
| Aa02 | **F:** CTGCAGATTTCACCTGTTCTG | GT_19_ | 128–146 | Spanish imperial eagle *(Aquila adalberti)* | Martínez-Cruz *et al.*, 2002 | 0.25 | VIC |
|  | **R:** CTTCCAGGTCTTGCAGTTTACC |  |  |  |  | 0.25 |  |
| Aa04 | **F:** TGCAGCTCAAAAGCAAAGG | GT_12_ | 117–159 | Spanish imperial eagle *(A. adalberti)* | Martínez-Cruz *et al.*, 2002 | 0.25 | NED |
|  | **R:** CAACCCCAACTCTCACACCT |  |  |  |  | 0.25 |  |
| Aa11 | **F:** ACGAGCTTATCTTTGACCAAGC | CA_11_ | 239–279 | Spanish imperial eagle *(A. adalberti)* | Martínez-Cruz *et al.*, 2002 | 0.25 | VIC |
|  | **R:** CTTTGTTTCAGCTGTTCCAGG |  |  |  |  | 0.25 |  |
| Aa15 | **F:** TCACTGACCTGCCCTCTACA | CA_13_ | 194–208 | Spanish imperial eagle *(A. adalberti)* | Martínez-Cruz *et al.*, 2002 | 0.125 | FAM |
|  | **R:** CCAACCCTCTAGTCGTCCAC |  |  |  |  | 0.125 |  |
| Aa26 | **F:** GCAAAGGTAAACTGCATCTGG | AC_14_ | 139–155 | Spanish imperial eagle *(A. adalberti)* | Martínez-Cruz *et al.*, 2002 | 0.125 | PET |
|  | **R:** ATGCACTATTGGTAAACAGGCA |  |  |  |  | 0.125 |  |
| Aa27 | **F:** GAGATGTCTTCACAGCTTGGC | CA_11_ | 84–100 | Spanish imperial eagle *(A. adalberti)* | Martínez-Cruz *et al.*, 2002 | 0.125 | VIC |
|  | **R:** AAGTCTCAGAGACTGACGGACC |  |  |  |  | 0.125 |  |
| Aa35 | **F:** GCAGCAGAAAGTGCATACGA | AC_17_ | 231–267 | Spanish imperial eagle *(A. adalberti)* | Martínez-Cruz *et al.*, 2002 | 0.25 | FAM |
|  | **R:** GACCAAATGAAATGCGCC |  |  |  |  | 0.25 |  |
| Aa36 | **F:** ACAGGCCAGCACCAAGAG | AC_16_ | 88–132 | Spanish imperial eagle *(A. adalberti)* | Martínez-Cruz *et al.*, 2002 | 0.25 | PET |
|  | **R:** TTTGGAGCCATTGTTACCGT |  |  |  |  | 0.25 |  |
| Aa39 | **F:** TTCTGTTTTTCCACTTGCTTG | AC_13_ | 182–200 | Spanish imperial eagle *(A. adalberti)* | Martínez-Cruz *et al.*, 2002 | 0.60 | VIC |
|  | **R:** TATTGAGCTCACAAAAACAAAGG |  |  |  |  | 0.60 |  |
| Aa43 | **F:** CCACACTGAGAAACTCCTGTTG | AC_14_ | 103–129 | Spanish imperial eagle *(A. adalberti)* | Martínez-Cruz *et al.*, 2002 | 0.125 | FAM |
|  | **R:** TTCCTGAGAGCTCTTCCTGC |  |  |  |  | 0.125 |  |
| NVHfr142 | **F:** CCACCCCTCTGCCACTCA | GT_12_ | 173–193 | Gyrfalcon  *(Falco rusticolus)* | Nesje & Roed, 2000 | 0.25 | PET |
|  | **R:** CCCCTGTCAGCTAAACACATCAC |  |  |  |  | 0.25 |  |
| NVHfr206 | **F:** ATCTAATGGGCTTTCCTGGATTT | CA_14_ | 153–169 | Gyrfalcon  *(F. rusticolus)* | Nesje & Roed, 2000 | 0.25 | FAM |
|  | **R:** GACATTTTCCTCATAGGCAACTGA |  |  |  |  | 0.25 |  |

**Table S3.** Accession numbers to GenBank sequences that were added to this study. The table shows haplotype names following GenBank, sequence lengths in base pairs, GenBank accession numbers, and references to the original studies.

| **Haplotype** | **Length, bp** | **Accession numbers** | **Reference** |
| --- | --- | --- | --- |
| CR1-CR4 | 446 | JQ246417-JQ246421 | Sonsthagen *et al.*, 2012 |
| H1-H17 | 402 | KR259260-KR259276 | Nebel *et al.*, 2015 |
| M1-M9 | 402 | KR259251-KR259259 | Nebel *et al.*, 2015 |
| GOEA01-GOEA07 | 415 | KX687705-KX687711 | Craig *et al.*, 2016 |
| N1-N18 | 402 | MG491978-MG491987 | Judkins & van den Bussche, 2017 |
| EST-2-3, EST-5-8, EST-12-25 | 402 | MN105919-MN105938 | Nebel *et al.*, 2019 |
| FIN1-FIN17 | 393 | OQ679875-OQ679891 | Kylmänen *et al.*, 2023 |

**Table S4.** Summary of the number of samples used for different mtDNA analyses with and without the GenBank data. The table shows the analysis, alignment length of the sequences used for the analysis (in base pairs), and number of samples included from this study (newly sequenced individuals) and the GenBank sequences, and the total sample size per analysis.

| **Analysis and alignment length (bp)** | **EURASIA** | | | | **NORTH AMERICA** | | | | **TOTAL** |
| --- | --- | --- | --- | --- | --- | --- | --- | --- | --- |
|  | **This study** | **Kylmänen *et al.* 2023** | **Nebel *et al.* 2015** | **Nebel *et al.* 2019** | **Nebel *et al.* 2015** | **Sonsthagen *et al.* 2012** | **Craig *et al.* 2016** | **Judkins & van den Bussche 2017** |  |
| Genetic diversity, geographical groups  (390 bp; main dataset) | 82 | 5 | - | - | - | - | - | - | 87 |
| Genetic diversity and ΦST, temporal groups  (390 bp; main dataset) | 72 | 5 | - | - | - | - | - | - | 77 |
| AMOVA and ΦST, geographical groups  (390 bp; main dataset) | 82 | 5 | - | - | - | - | - | - | 87 |
| Genetic diversity, geographical groups  (326 bp) | 82 | 88 | 244 | 20 | - | - | - | - | 434 |
| Haplotype Network  (326 bp) | 82 | 88 | 244 | 20 | 5 | 5 | 49 | 170 | 663 |
| AMOVA and ΦST, demographic tests  (326 bp) | 82 | 88 | 244 | 20 | - | - | - | - | 434 |
| Temporal analyses  (326 bp) | 72 | 88 | 233 | - | - | - | - | - | 393 |

**Table S5.** Summary of how samples from this study and GenBank were assigned to five geographical groups. The table shows the number of sequences per reference (N); the countries (or regions) from where the sequences were obtained; and references to the original studies. Sample size for Northern Europe in this study is presented for both, newly sampled individuals (N = 14), and when the five Finnish golden eagles were added from Kylmänen *et al.* (2023; N = 19).

| **Geographical group** | **N** | **Country** | **Reference** |
| --- | --- | --- | --- |
| Northern Europe | 14(19)* | Russia (Northwestern federal district), Sweden | This study |
|  | 88 | Finland | Kylmänen *et al.* 2023 |
|  | 20 | Estonia | Nebel *et al.* 2019 |
|  | 51 | Finland, Norway, Scotland | Nebel *et al.* 2015 |
| Central and Eastern Europe | 39 | Russia (Central and Volga federal districts), Austria, Belarus, Ukraine | This study |
|  | 156 | Alps, Austria, Bulgaria, Greece, Hungary, Italy, Romania, Russia (European), Ukraine | Nebel *et al.* 2015 |
| Central Asia and Caucasus | 18 | Russia (North Caucasian federal district), Azerbaijan, Iran, Kazakhstan, Kyrgyzstan, Uzbekistan, Turkmenistan | This study |
|  | 4 | Turkey, Afghanistan, unknown country in Central Asia | Nebel *et al.* 2015 |
| Far East | 15 | Russia (Siberian and Far Eastern federal districts) | This study |
|  | 17 | Russia (Asian), China, Japan | Nebel *et al.* 2015 |
| Western Europe | 16 | Spain, non-Alpine France | Nebel *et al.* 2015 |

**Table S6**. Calculation of golden eagle’s replacement (generation) time. Country – country for which the calculation was done; years of study – period of when the reference study was conducted; N young/pair/year – number of chicks born per pair per year; mortality rate, non-adult – mortality rate of golden eagles before reaching sexual maturity, survival rate, non-adult – survival rate of golden eagles before reaching sexual maturity (1 – mortality rate, non-adult); reference – reference to the study where the breeding performance and mortality rates were published; N chick/pair – number of chicks per pair that reach sexual maturity (N young/pair/year × survival rate), replacement time – time in years that takes two adults to replace themselves (= 2/ number of chicks per pair that will reach sexual maturity); average age of wild adults (replacement time + 4.5 years; 4.5 was taken as the average age of golden eagles reaching sexual maturity).

| **Country** | **Year(s) of the study** | **N young/pair/year** | **Mortality rate, non-adult** | **Survival rate, non-adult** | **Reference** |  | **N chicks/pair that reach sexual maturity** | **Replacement time, years** | **Average age of wild adults, years** |
| --- | --- | --- | --- | --- | --- | --- | --- | --- | --- |
| Finland | 2018 | 0.37 | 0.77 | 0.23 | Ollila, 2019 |  | 0.09 | 23.50 | 28.00 |
| Finland | 1971 | 0.72 | 0.77 | 0.23 | Ollila, 2019 |  | 0.17 | 12.08 | 16.58 |
| Finland | 1960-1970 | 0.48 | 0.77 | 0.23 | Sulkava *et al.*, 1984 |  | 0.11 | 18.12 | 22.62 |
| Finland | 1971-1977 | 0.54 | 0.77 | 0.23 | Sulkava *et al.*, 1984 |  | 0.12 | 16.10 | 20.60 |
| Sweden | 1970 | 0.64 | 0.77 | 0.23 | Sulkava *et al.*, 1984 |  | 0.15 | 13.59 | 18.09 |
| Scotland | 1982-1985 | 0.56 | 0.61 | 0.39 | Whitfield *et al.*, 2004 |  | 0.22 | 9.14 | 13.64 |
| Scotland, Sutherland | 1967 | 0.4 | 0.61 | 0.39 | Whitfield *et al.*, 2004 |  | 0.16 | 12.80 | 17.30 |
| Scotland | 2003 | 0.46 | 0.61 | 0.39 | Whitfield *et al.*, 2004 |  | 0.18 | 11.13 | 15.63 |
|  | | | | | | MINIMUM | 0.09 | **9.14** | 13.64 |
|  |  |  |  |  |  | MAXIMUM | 0.22 | **23.50** | 28.00 |
|  |  |  |  |  |  | MEAN | 0.15 | **14.56** | 19.06 |

**Table S7.** Summary table of the Tracer output for the BEAST analyses of Eurasian golden eagles: Total, Holarctic, Mediterranean with the number of sequences used and an indication of the number of independent runs needed to achieve effective sample size (ESS) > 200. Each run was performed with 100 million MCMC and a 10% burn-in. We corrected three operators according to BEAST recommendations from the first run: scale in UpDown operator of strict clock was set to 0.58, delta in delta exchange operator was set to 72 028.879, and scale factor in Bayesian skyline tree scaler was set to 0.789. Statistics – the name of the trace statistics, mean - the mean value of the sampled trace across the chain excluding the burn-in, ESS – the number of independent samples that achieved the trace.

| **Statistic** | **Total (N = 393),**  **8 runs** | | **Holarctic (N = 249),**  **6 runs** | | **Mediterranean (N = 144),**  **2 runs** | |
| --- | --- | --- | --- | --- | --- | --- |
|  | **Mean** | **ESS** | **Mean** | **ESS** | **Mean** | **ESS** |
| posterior | -4321.41 | 266 | -3234.84 | 249 | -1580.73 | 222 |
| likelihood | -864.60 | 846 | -739.22 | 1107 | -570.06 | 578 |
| prior | -3456.81 | 266 | -2495.62 | 245 | -1010.67 | 216 |
| treeLikelihood | -864.60 | 846 | -739.22 | 1107 | -570.06 | 578 |
| TreeHeight | 10574.85 | 650 | 9010.76 | 830 | 803.57 | 449 |
| clockRate | 8.12E-06 | 610 | 5.40E-06 | 473 | 1.51E-05 | 358 |
| mutationRate | 1 | - | 1 | - | 35.23 | 4650 |
| kappa | 73.63 | 24602 | 72.99 | 16672 | 1.00 | - |
| BayesianSkyline | -3405.81 | 267 | -2438.31 | 245 | -969.85 | 216 |
| bPopSizes.1 | 422.44 | 739 | 19882.02 | 2634 | 490.57 | 1004 |
| bPopSizes.2 | 812.67 | 284 | 13999.98 | 1029 | 429.77 | 362 |
| bPopSizes.3 | 1933.76 | 302 | 14829.44 | 616 | 638.51 | 383 |
| bPopSizes.4 | 3858.78 | 294 | 13380.77 | 536 | 833.91 | 429 |
| bPopSizes.5 | 4000.40 | 538 | 5196.67 | 930 | 489.71 | 582 |
| bGroupSizes.1 | 34.58 | 342 | 53.18 | 7921 | 32.02 | 4798 |
| bGroupSizes.2 | 25.58 | 360 | 55.32 | 13349 | 31.53 | 14573 |
| bGroupSizes.3 | 54.41 | 541 | 56.50 | 15386 | 29.21 | 15064 |
| bGroupSizes.4 | 101.69 | 507 | 49.59 | 10932 | 27.67 | 11503 |
| bGroupSizes.5 | 175.73 | 261 | 33.41 | 6047 | 22.58 | 4921 |
| freqParameter.1 | 0.26 | 22875 | 0.25 | 20618 | 0.25 | 9049 |
| freqParameter.2 | 0.24 | 20610 | 0.24 | 19232 | 0.24 | 8835 |
| freqParameter.3 | 0.20 | 30391 | 0.21 | 27537 | 0.20 | 8760 |
| freqParameter.4 | 0.29 | 30069 | 0.30 | 23625 | 0.30 | 8721 |

**Table S8.** Summary statistics on 12 microsatellite loci used in genotyping 91 golden eagles. N – number of individuals genotyped for the locus; ADO – allele dropout rate per all loci; FA – false allele rate per all loci; NULL – null allele frequency; A – number of alleles; PIC – polymorphic information content; AR – allelic richness; H_O_ – observed heterozygosity; H_E_ – unbiased expected heterozygosity; F_IS_ – inbreeding coefficient; SE – standard error.

| **Locus** | **N** | **Genotyping success** | **Total error rate per allele** | **ADO** | **FA** | **Total error rate per all loci** | **NULL** | **A** | **PIC** | **AR** | **H_O_** | **H_E_** | **F_IS_** |
| --- | --- | --- | --- | --- | --- | --- | --- | --- | --- | --- | --- | --- | --- |
| Aa02 | 90 | 98.9 % | 0.037 | 0.036 | 0.030 | 0.065 | 0.061 | 6 | 0.47 | 5.72 | 0.456 | 0.530 | 0.142 |
| Aa04 | 91 | 100 % | 0.070 | 0.073 | 0.051 | 0.124 | 0.078 | 13 | 0.73 | 11.26 | 0.626 | 0.767 | 0.184 |
| Aa11 | 61 | 67.0 % | 0.025 | 0.081 | 0.010 | 0.091 | 0.083 | 10 | 0.59 | 9.33 | 0.492 | 0.634 | 0.225 |
| Aa15 | 84 | 92.3 % | 0.028 | 0.046 | 0.007 | 0.052 | 0.042 | 6 | 0.44 | 5.91 | 0.417 | 0.465 | 0.104 |
| Aa26 | 89 | 97.8 % | 0.037 | 0.054 | 0.012 | 0.065 | 0.013 | 5 | 0.63 | 5.00 | 0.674 | 0.690 | 0.024 |
| Aa27 | 91 | 100 % | 0.037 | 0.051 | 0.017 | 0.067 | 0.053 | 4 | 0.56 | 3.77 | 0.560 | 0.634 | 0.117 |
| Aa35 | 47 | 51.6 % | 0.014 | 0.069 | 0.000 | 0.069 | 0.166 | 9 | 0.74 | 9.00 | 0.489 | 0.779 | 0.375 |
| Aa36 | 79 | 86.8 % | 0.073 | 0.093 | 0.053 | 0.146 | 0.113 | 11 | 0.79 | 6.37 | 0.620 | 0.817 | 0.293 |
| Aa39 | 76 | 83.5 % | 0.034 | 0.055 | 0.023 | 0.078 | 0.141 | 11 | 0.78 | 9.95 | 0.539 | 0.805 | 0.331 |
| Aa43 | 89 | 97.8 % | 0.034 | 0.017 | 0.035 | 0.052 | 0 | 8 | 0.68 | 7.05 | 0.719 | 0.717 | -0.003 |
| NVHfr142 | 72 | 79.1 % | 0.039 | 0.085 | 0.025 | 0.110 | 0.165 | 8 | 0.64 | 7.85 | 0.444 | 0.691 | 0.359 |
| NVHfr206 | 89 | 97.8 % | 0.008 | 0.012 | 0.006 | 0.018 | 0 | 2 | 0.06 | 1.99 | 0.067 | 0.066 | -0.029 |
| **All loci mean** | **79.83** | **87.6 %** | **0.036** | **0.056** | **0.022** | **0.078** | **0.076** | **7.75** | **0.59** | **6.93** | **0.509** | **0.633** | **0.18** |
| SE | 3.85 | 0.04 | 0.005 | 0.007 | 0.005 | 0.010 | 0.016 | 0.90 | 0.06 | 0.74 | 0.047 | 0.058 | 0.04 |


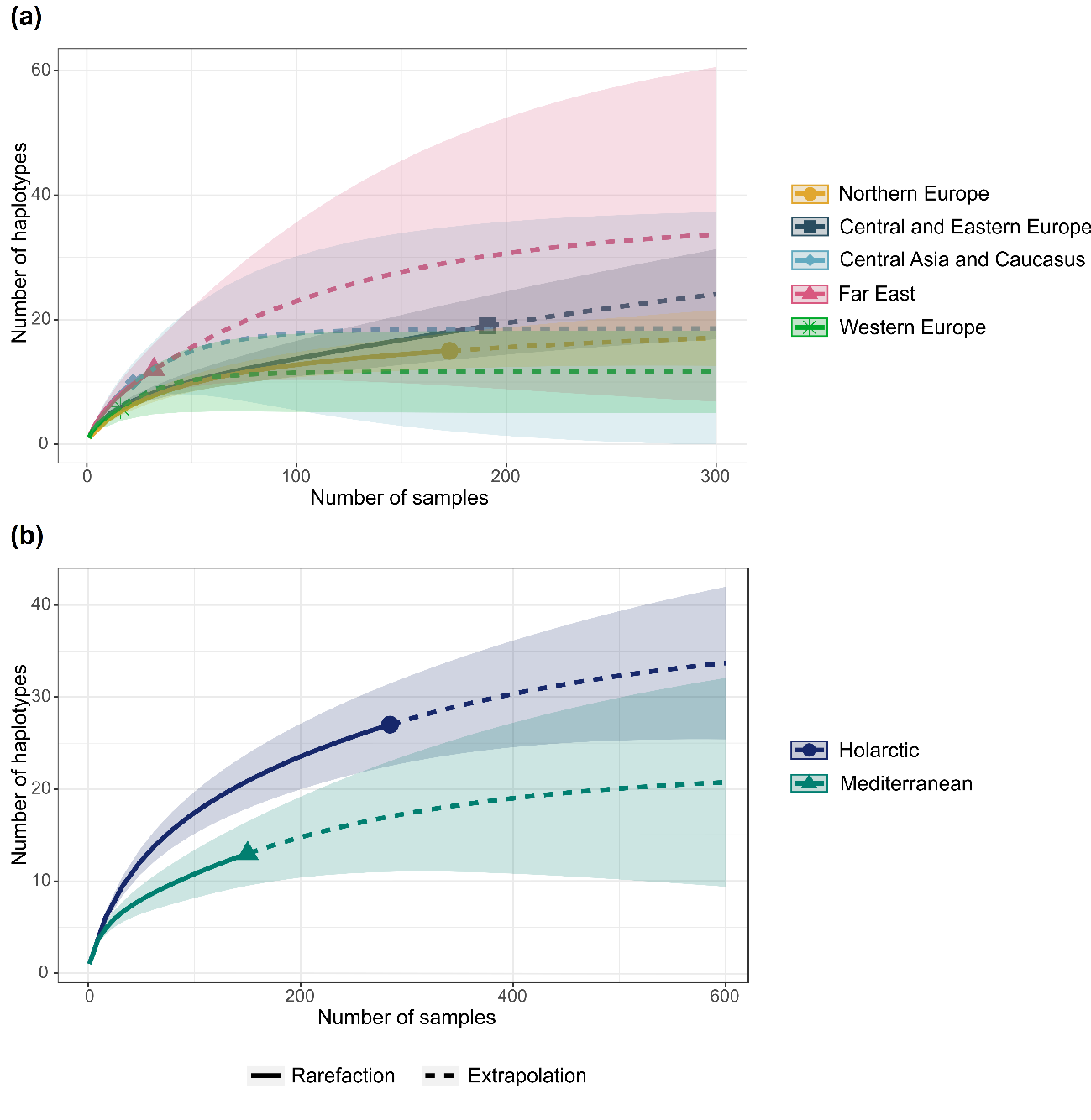


**Figure S2**. Results of rarefaction-extrapolation analyses (iNEXT) for the number of haplotypes found in different groups of Eurasian golden eagles. The analyses were performed including sequences from GenBank. **(a)** Comparison between five geographical groups (N = 434). **(b)** Comparison between Mediterranean and Holarctic groups (N = 434).

**Table S9.** Mitochondrial genetic diversity of 87 Eurasian golden eagles using only our data (i.e., the main dataset of 82 sequences from this study and five added from Kylmänen *et al.* (2023); CR, 390 bp). N – number of individuals; H – number of haplotypes; h – haplotype diversity; π – nucleotide diversity; θ_W_ (S) – Watterson’s theta (mutation parameter theta per site) calculated based on the number of segregating sites.

| **Group** | **N** | **H** | **h** | **π** | **θ_W_ (S)** |
| --- | --- | --- | --- | --- | --- |
| Northern Europe | 19 | 7 | 0.608 | 0.0024 | 0.0051 |
| Central and Eastern Europe | 35 | 9 | 0.620 | 0.0038 | 0.0106 |
| Central Asia and Caucasus | 18 | 8 | 0.752 | 0.0141 | 0.0104 |
| Far East | 15 | 6 | 0.571 | 0.0045 | 0.0071 |
| Mediterranean | 8 | 5 | 0.857 | 0.0035 | 0.0040 |
| Holarctic | 79 | 18 | 0.560 | 0.0028 | 0.0078 |
| Bottleneck (1885-1984) | 64 | 17 | 0.587 | 0.0066 | 0.0103 |
| Post-bottleneck (1989-2017) | 13 | 8 | 0.859 | 0.0042 | 0.0074 |
| EURASIA | 87 | 23 | 0.636 | 0.0067 | 0.0112 |

**Table S10.** 23 golden eagle haplotypes found in this study. Here we present haplotype names and frequencies (number of golden eagles with a particular haplotype), countries of occurrences (and for Russia – regions), and the accession numbers under which the sequences were submitted to GenBank. Also, we report a fully matching haplotype from our previous study of Finnish golden eagles (FIN, Kylmänen *et al.*, 2023) in a 390 bp alignment; and the amalgamated haplotypes, i.e., haplotypes from previous studies to which haplotypes of this study match in a 326 bp alignment. CR – North American (Sonsthagen *et al.*, 2012), H – Holarctic (Nebel *et al.*, 2015), M – Mediterranean (Nebel *et al.*, 2015), N – North American (Judkins & van den Bussche, 2017), and EST – Estonian (Nebel *et al.*, 2019). The alignment to GenBank sequences resulted in a loss of two single nucleotide polymorphism positions, masking the divergence between some haplotypes found in this study: RUS6, RUS12, and RUS17 are amalgamated to H1. Eight haplotypes (RUS1-3, IRN1, KAZ1, KYR1-2, UZB1) were newly discovered.

| **Haplotype, this study, 390 bp** | **Haplotype frequency** | **Country (and region) of occurrence** | **GenBank accession number** | **Full match to FIN haplotype, 390 bp alignment** | **Amalgamated haplotype, 326 bp alignment** | **Code in a haplotype network** |
| --- | --- | --- | --- | --- | --- | --- |
| RUS1 | 1 | Russia: Kamchatka | OR635080 | - | - | RUS1 |
| RUS2 | 1 | Russia: Kamchatka | OR635081 | - | - | RUS2 |
| RUS3 | 1 | Russia: Bryansk oblast | OR635082 | - | - | RUS3 |
| RUS4 | 1 | Russia: Karelia | OR635083 | FIN5 | - | FIN5 |
| RUS5 | 1 | Russia: Komi | OR635084 | FIN3 | H12 | H12 |
| RUS6 | 1 | Russia: Sverdlovsk oblast | OR635085 | - | H1 | H1 |
| RUS7 | 1 | Russia: Komi | OR635086 | FIN9 | N12 | N12 |
| RUS8 | 1 | Russia: Kamchatka | OR635087 | FIN14 | EST-18 | EST-18 |
| RUS9 | 1 | Russia: Adygea | OR635088 | - | H16 | H16 |
| RUS10 | 1 | Russia: Kamchatka | OR635089 | - | H8 | H8 |
| RUS11 | 1 | Russia: Jewish autonomous oblast | OR635090 | - | CR4 | CR4 |
| RUS12 | 1 | Russia: Kirov oblast | OR635091 | FIN11 | H1 | H1 |
| RUS13 | 1 | Russia: Kirov oblast | OR635092 | FIN17 | - | FIN17 |
| RUS14 | 2 | Russia: Komi and Kirov oblast | OR635093 | FIN13 | H14 | H14 |
| RUS15 | 2 | Russia: Kirov oblast, Moscow zoo | OR635094 | FIN8 | H2 | H2 |
| RUS16 | 7 | Russia: Bashkortostan, Komi, Novgorod oblast, Kirov oblast; Belarus | OR635095 | FIN2 | H4 | H4 |
| RUS17 | 49 | Austria, Azerbaijan, China, Kazakhstan, Kyrgyzstan, Russia, Sweden, Switzerland, Turkmenistan, Ukraine | OR635096 | FIN1 | H1 | H1 |
| IRN1 | 1 | Iran | OR635097 | - | - | IRN1 |
| KAZ1 | 1 | Kazakhstan | OR635098 | - | - | KAZ1 |
| KAZ2 | 3 | Kazakhstan, Turkmenistan, Austria | OR635099 | FIN7 | M1 | M1 |
| KYR1 | 1 | Kyrgyzstan | OR635100 | - | - | KYR1 |
| KYR2 | 2 | Russia: Northern Osetiya; Kyrgyzstan | OR635101 | - | - | KYR2 |
| UZB1 | 1 | Uzbekistan | OR635102 | - | - | UZB1 |

**Table S11.** Results of the Mann-Whitney U test (Wilcoxon rank sum test) and the Kruskal-Wallis tests for comparing genetic diversity estimates among groups of 91 Eurasian golden eagles. A – number of alleles, H_O_ – observed heterozygosity, H_E_ – expected heterozygosity, F_IS_ – inbreeding coefficient. Asterisk indicates the statistically significant result.

| Compared groups | Statistical result | | | |
| --- | --- | --- | --- | --- |
|  | A | H_O_ | H_E_ | F_IS_ |
| Mitochondrial lineage: Mediterranean VS Holarctic | W = 104,  p = 0.065 | W = 63,  p = 0.623 | W = 54.5,  p = 0.326 | W = 86,  p = 0.436 |
| Temporal groups: Bottleneck VS Post-bottleneck | W = 36.5,  p = 0.400 | W = 71,  p = 0.977 | W = 71.5,  p = 1 | W = 44,  p = 0.293 |
| Geographical groups:   - Northern Europe - Central and Eastern Europe - Central Asia and Caucasus - Far East | Kruskal-Wallis chi-squared = 1.5345,  df = 3,  p = 0.674 | Kruskal-Wallis chi-squared = 3.74,  df = 3,  p = 0.291 | Kruskal-Wallis chi-squared = 1.254,  df = 3,  p = 0.740 | Kruskal-Wallis chi-squared = 5.117,  df = 3,  p = 0.163 |
| Northern Europe VS Central and Eastern Europe | W = 66,  p = 0.747 | W = 82.5,  p = 0.564 | W = 71,  p = 0.977 | W = 45.4,  p = 0.341 |
| Northern Europe VS Central Asia and Caucasus | W = 62.5,  p = 0.598 | W = 74,  p = 0.931 | W = 56,  p = 0.371 | W = 47,  p = 0.393 |
| Northern Europe VS Far East | W = 83,  p = 0.538 | W = 49,  p = 0.194 | W = 64,  p = 0.665 | W = 79,  p = 0.237 |
| Central and Eastern Europe VS Central Asia and Caucasus | W = 68.5,  p = 0.861 | W = 67,  p = 0.795 | W = 55,  p = 0.341 | W = 64,  p = 0.844 |
| Central and Eastern Europe VS Far East | W = 90,  p = 0.306 | W = 36.5,  p = 0.043* | W = 64.5,  p = 0.686 | W = 90,  p = 0.057 |
| Central Asia and Caucasus VS Far East | W = 89.5,  p = 0.321 | W = 54,  p = 0.312 | W = 80.5,  p = 0.644 | W = 87,  p = 0.088 |


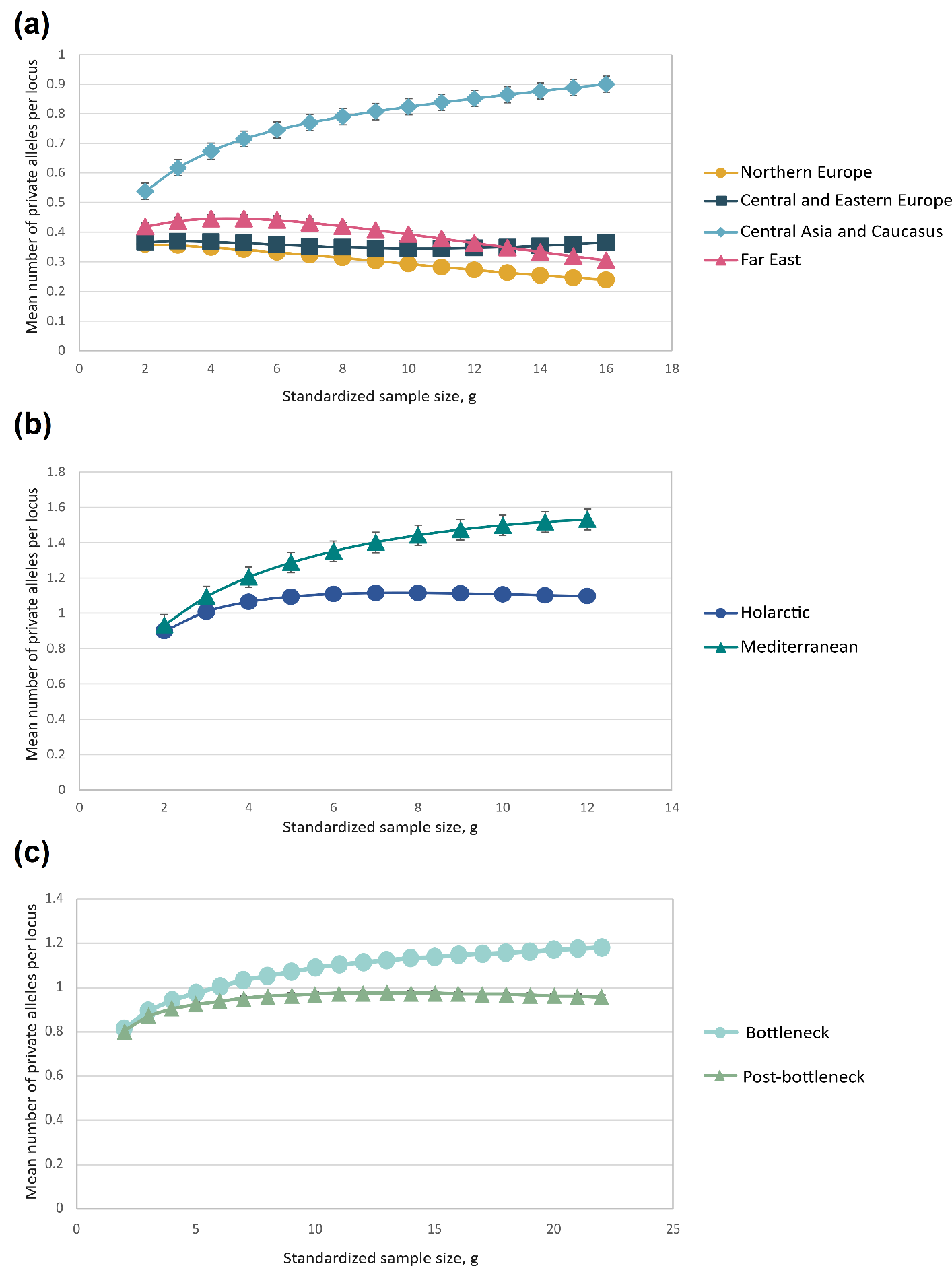


**Figure S3.** The mean private allelic richness (number of private alleles per locus) as a function of standardized sample size for the 91 Eurasian golden eagles divided into three groups. **(a)** The four geographical groups. **(b)** Mediterranean and Holarctic lineages. **(c)** Temporal groups: Bottleneck (1885-1984) and Post-bottleneck (1985-2017). Standard error bars are included but are not visible for each data point due to small values.

**Table S12.** Pairwise ɸ_ST_ values for the four geographical groups (Northern Europe, Central and Eastern Europe, Central Asia and Caucasus, and Far East) of Eurasian golden eagles using only our data (N = 87). The ɸ_ST_ were calculated with Kimura 2-parameter distance model. P-values after 10 000 permutations are in parentheses.

|  | **Northern Europe** | **Central and Eastern Europe** | **Central Asia and Caucasus** | **Far East** |
| --- | --- | --- | --- | --- |
| **Northern Europe** | ― |  |  |  |
| **Central and Eastern Europe** | -0.012  (p = 0.650) | ― |  |  |
| **Central Asia and Caucasus** | 0.329  (p = 0.001) | 0.316  (p < 0.001) | ― |  |
| **Far East** | 0.015  (p = 0.255) | 0.015  (p = 0.221) | 0.242  (p = 0.008) | ― |

**Table S13.** Summary of the STRUCTURE runs of cluster assignment of 91 golden eagles using 12 microsatellite loci. For each run, the number of individuals is given, as well as the number of tested K, whether or not LOCPRIOR was used, the type of LOCPRIOR, and the results to estimate the optimal K using three methods: log probability (L(K)), Evanno’s ΔK, and Puechmaille’s Optimal K (MedMed(K)). In all runs, the burn-in period was 100 000, the number of MCMC repetitions was 500 000, and the number of iterations was 10.

| **Run** | **N** | **Tested K** | **LOCPRIOR** | **LOCPRIOR type** | **L(K)** | **ΔK (Evanno)** | **Optimal K, (Puechmaille)** |
| --- | --- | --- | --- | --- | --- | --- | --- |
| Total | 91 | 1-5 | No | - | 1 | 3 | - |
| Total | 91 | 1-5 | Yes | Geographical groups | 2 | 2 | 2-3 |
| Total, excluding Central Asia and Caucasus | 73 | 1-5 | Yes | Geographical groups | 1 | 2 | 2 |
| Individuals sequenced with mtDNA | 87 | 1-5 | Yes | Mitochondrial lineage: Mediterranean or Holarctic | 1 | 2 | 2-3 |
| Individuals with year information | 80 | 1-5 | Yes | Temporal group: Bottleneck or Post-bottleneck | 1 | 2 | 1-3 |


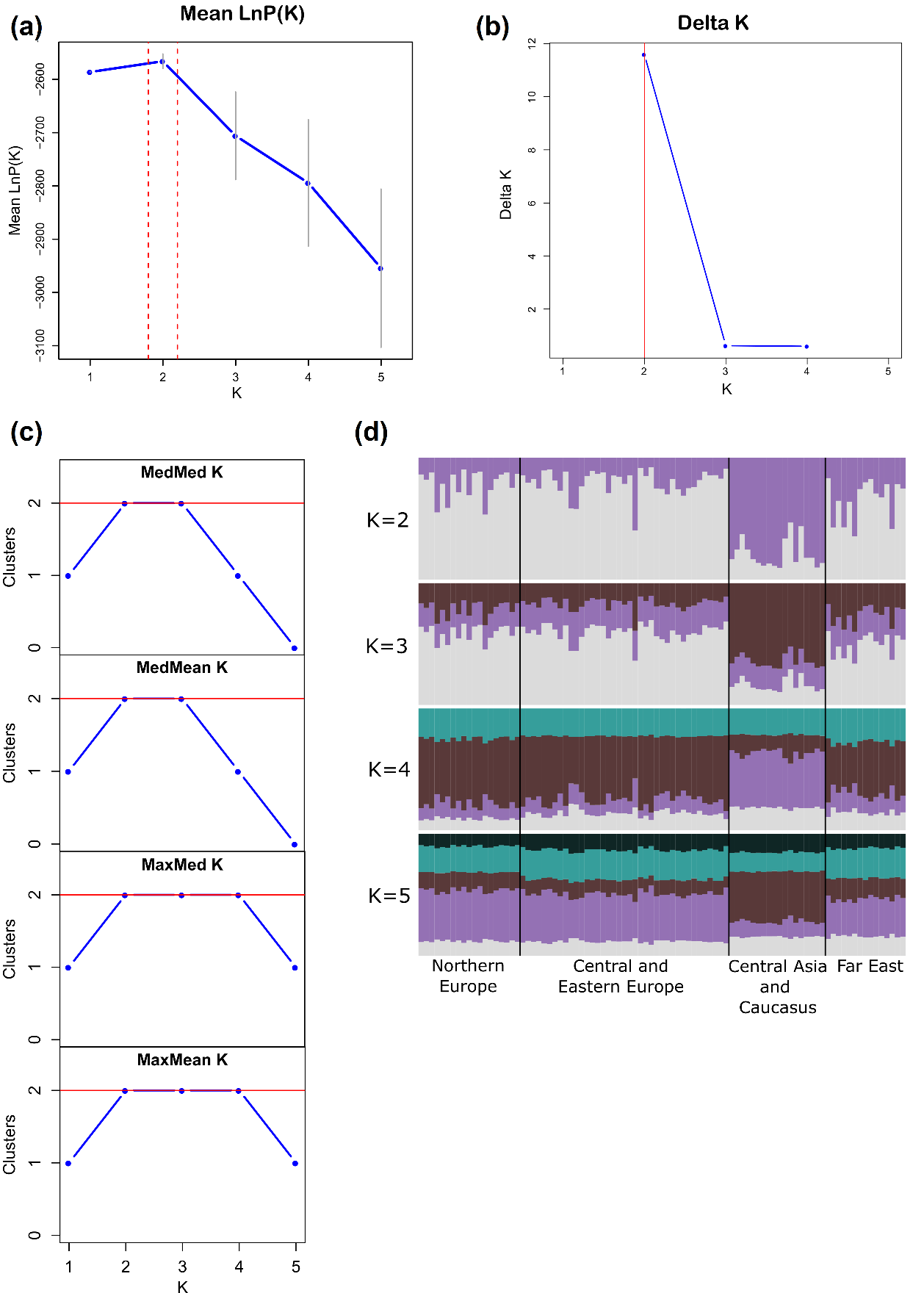


**Figure S4.** STRUCTURE results of cluster assignment of 91 golden eagles from the four geographical groups based on 12 microsatellite loci for K = 1 to 5. Colors represent genetic clusters. Each individual is presented as a bar, and the amount of each color indicates the proportion of each inferred cluster. **(a)** Log likelihood values (L(K)) for each tested K. **(b)** ΔK (Evanno’s method) for each tested K. **(c)** Optimal K (Puechmaille method): median of the median (MedMed K), median of the mean (MedMean K), maximum of the median (MaxMed K), and maximum of the mean (MaxMean K) for each tested K. **(d)** STRUCTURE assignment plots for each tested K with geographical groups as LOCPRIOR.


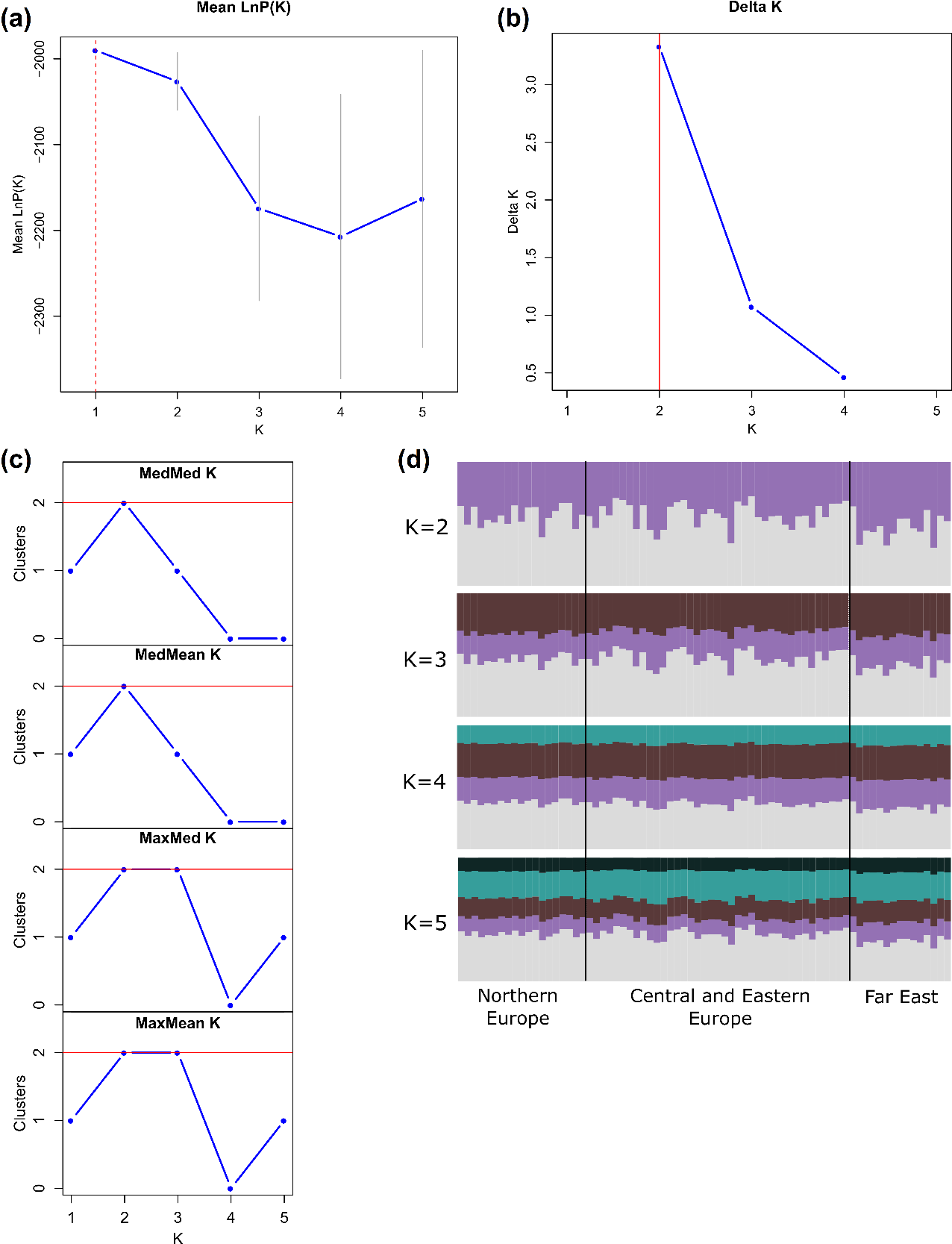


**Figure S5.** STRUCTURE results of cluster assignment of golden eagles excluding individuals from Central Asia and Caucasus. The results are for 73 golden eagles from the three geographical groups based on 12 microsatellite loci for K = 1 to 5. Colors represent genetic clusters. Each individual is presented as a bar, and the amount of each color indicates the proportion of each inferred cluster. **(a)** Log likelihood values (L(K)) for each tested K. **(b)** ΔK (Evanno’s method) for each tested K. **(c)** Optimal K (Puechmaille method): median of the median (MedMed K), median of the mean (MedMean K), maximum of the median (MaxMed K), and maximum of the mean (MaxMean K) for each tested K. **(d)** STRUCTURE assignment plots for each tested K with geographical groups as LOCPRIOR.


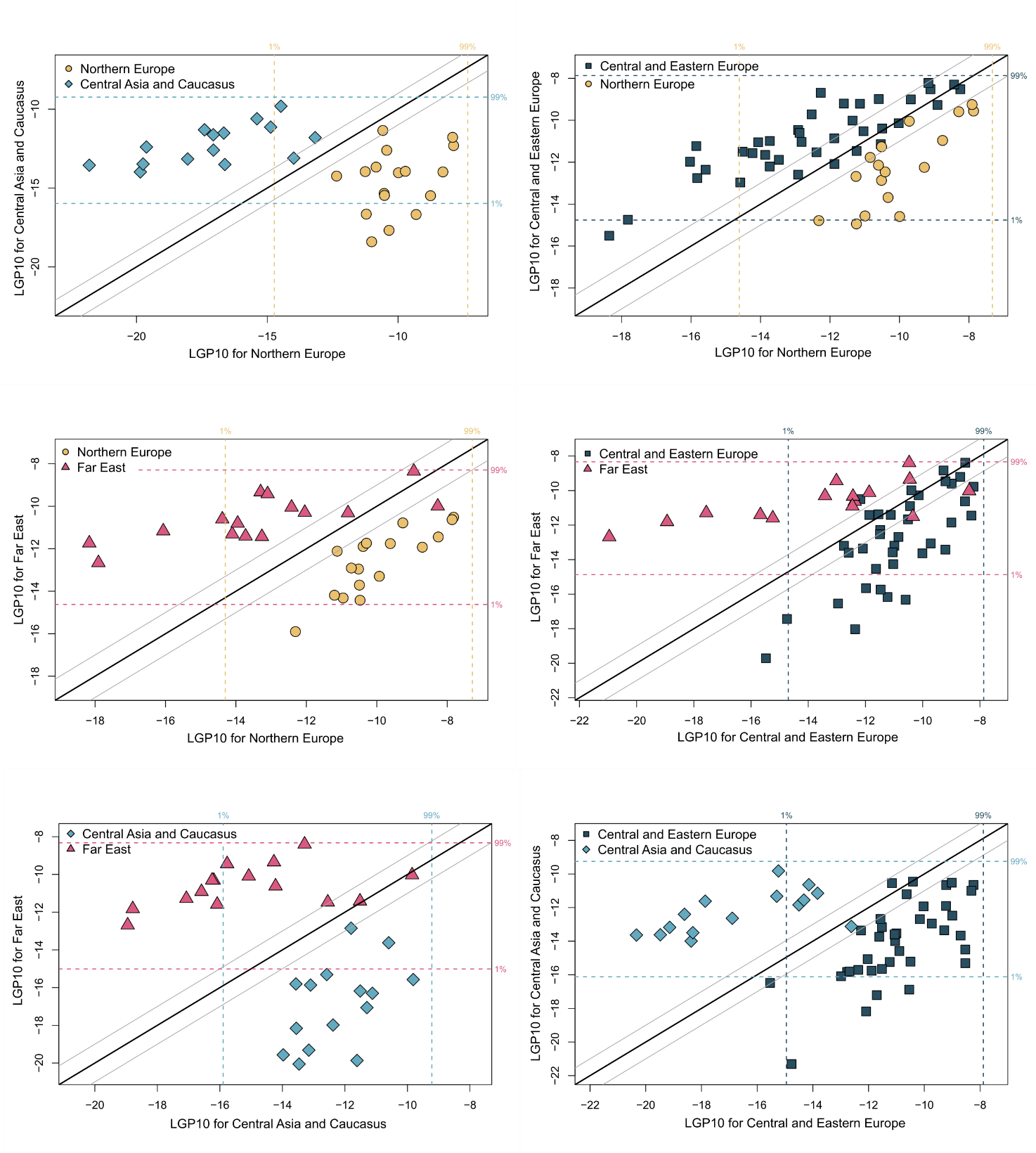


**Figure S6.** Geneplot results of pairwise comparisons of the four geographical groups of golden eagles with minimum of eight loci genotyped. The plots represent the log genotype probability (LGP10) values for assigning the individual to a chosen group. The range where genetic assignment of individuals into these groups is likely, is outlined within 1% and 99% quantiles.


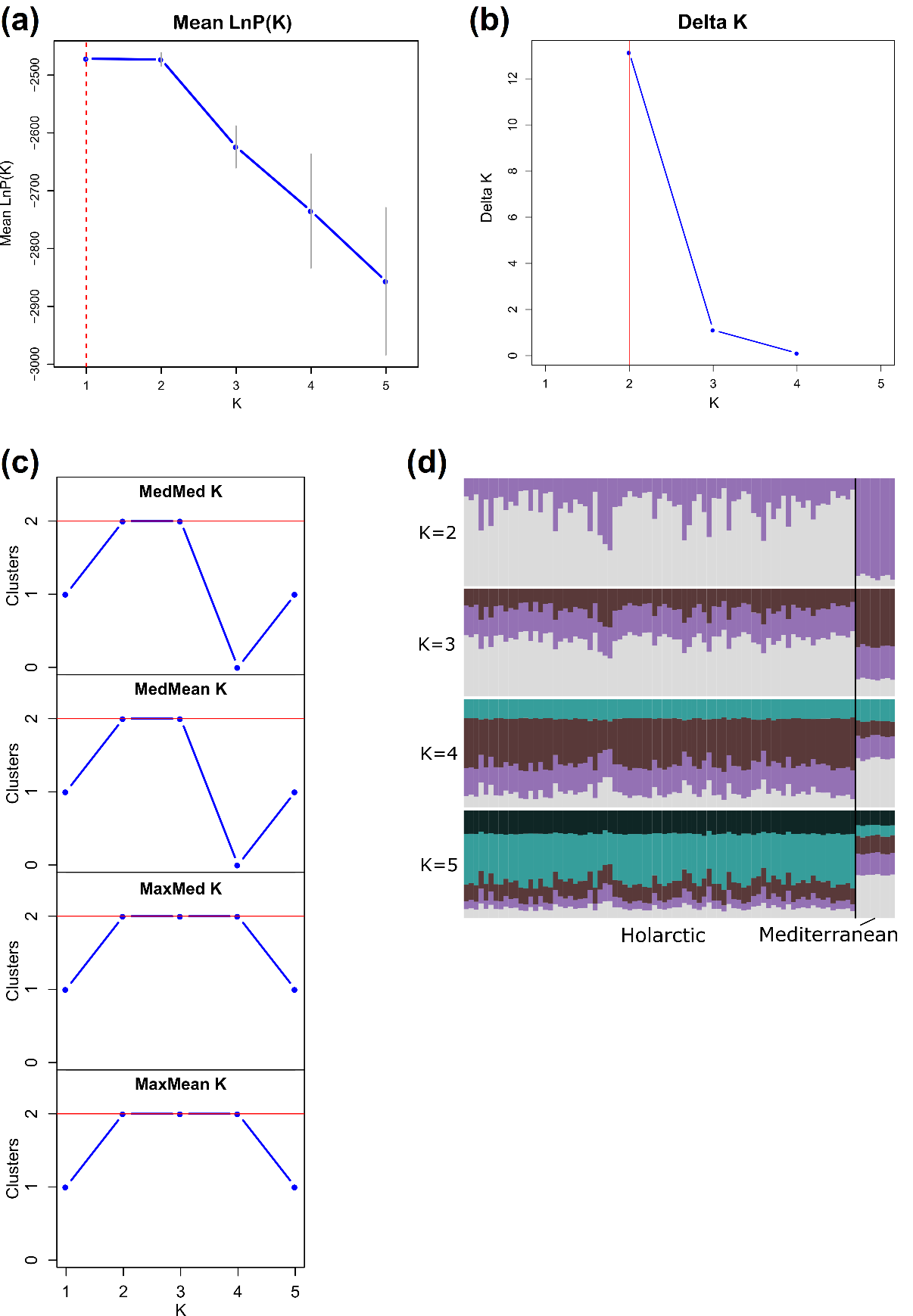


**Figure S7.** STRUCTURE results of cluster assignment of 87 golden eagles grouped according to the mitochondrial lineage (Holarctic and Mediterranean) using 12 microsatellite loci for K = 1 to 5. **(a)** Log likelihood (L(K)) values for each tested K. L(K) for K = 1 was -2471.8 and for K = 2 was -2473. **(b)** ΔK values (Evanno’s method) for each tested K. **(c)** Optimal K (Puechmaille method): median of the median (MedMed K), median of the mean (MedMean K), maximum of the median (MaxMed K), and maximum of the mean (MaxMean K) for each tested K. **(d)** STRUCTURE assignment plots for each tested K with mitochondrial lineages as LOCPRIOR. Colors represent genetic clusters. Each individual is presented as a bar, and the amount of each color indicates the proportion of each inferred cluster.


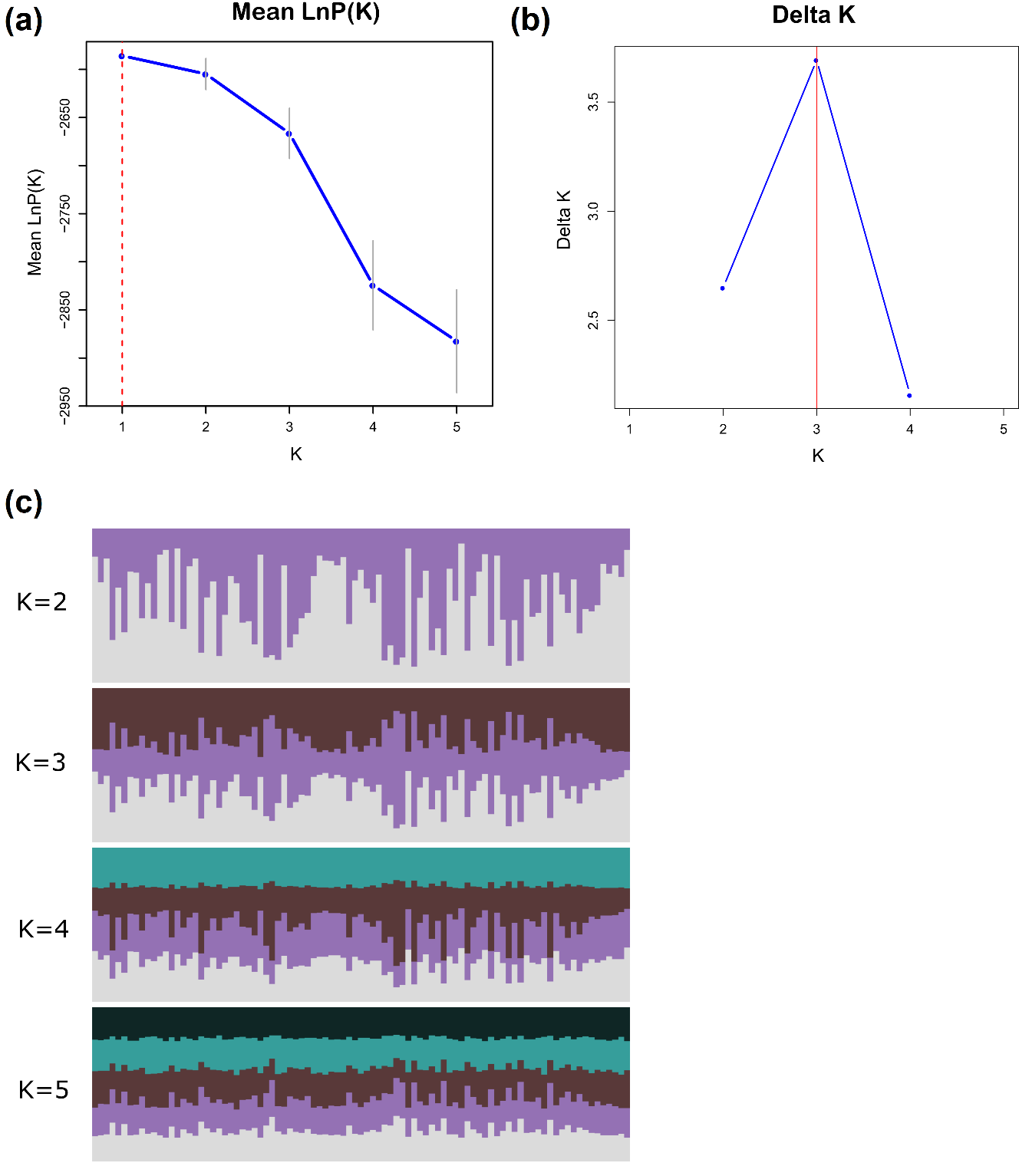


**Figure S8.** STRUCTURE results of cluster assignment of 91 golden eagles without LOCPRIOR information based on 12 microsatellite loci for K = 1 to 5. **(a)** Log likelihood (L(K)) values for each tested K. **(b)** ΔK values (Evanno’s method) for each tested K. **(c)** STRUCTURE assignment plots for each tested K. Colors represent genetic clusters. Each individual is presented as a bar, and the amount of each color indicates the proportion of each inferred cluster.


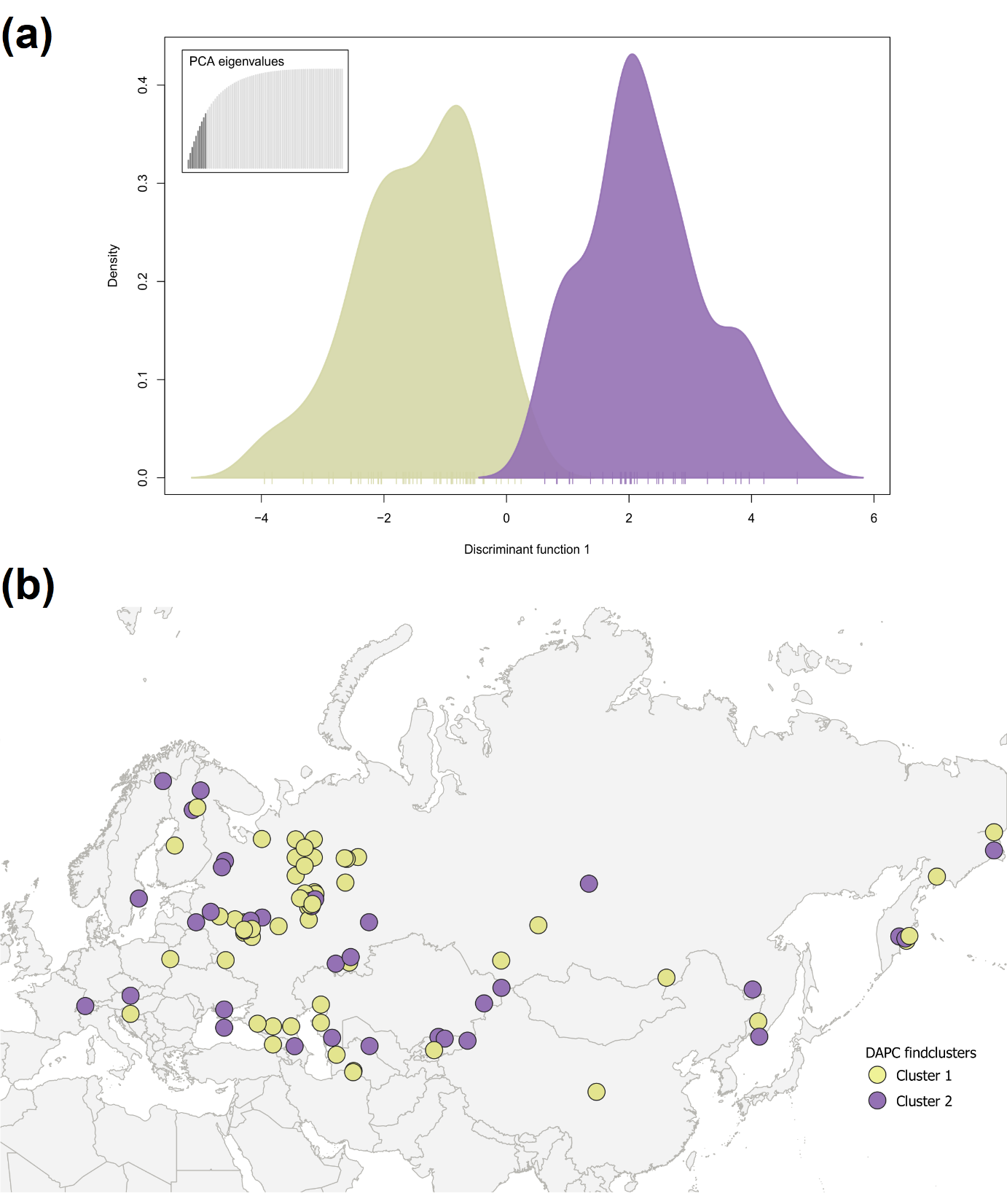


**Figure S9.** DAPC results of the *de novo* grouping of 91 golden eagles into two clusters (K = 2) using the find.clusters() command according to the lowest BIC value. Note that the assignment was done based on only 10 eigenvalues. **(a)** A one-dimensional DAPC plot based on ten principal components (PCs) accounting for 54.8% of variation. **(b)** Locations of individuals colored according to the cluster assignment based on the *de novo* grouping. The map was created in QGIS 3.10.


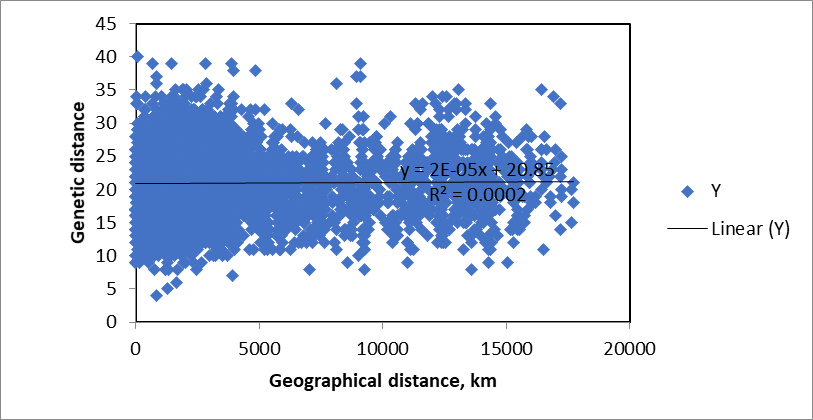


**Figure S10.** Mantel test for 91 Eurasian golden eagles using 12 microsatellites. Genetic distances were plotted against geographical distances in kilometers with 99 permutations. No isolation-by-distance (IBD) was found.


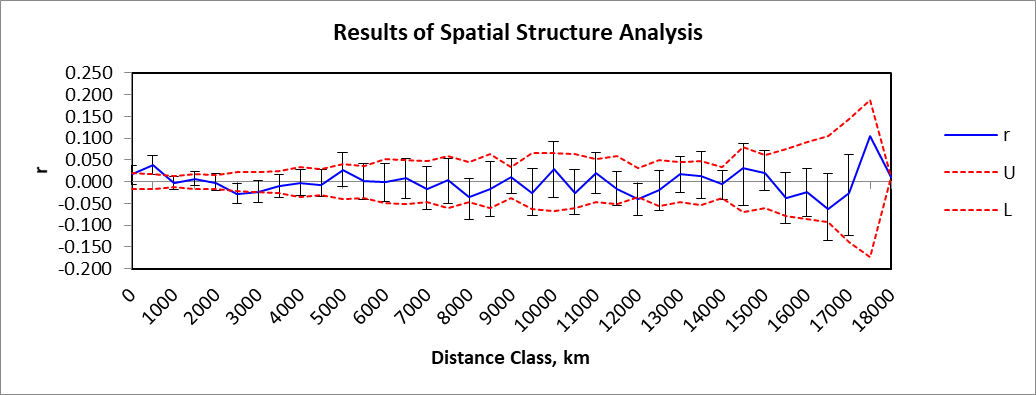


**Figure S11.** Correlogram of spatial autocorrelation analysis for 91 Eurasian golden eagles. Autocorrelation coefficient (r) is plotted against 37 equal distance classes (class size = 500) with upper (U) and lower (L) 95% confidence intervals after 1 000 Bootstrap replications. Correlogram is significant (Omega = 134.797, p = 0.002). No spatial correlation to genetic differentiation among individuals was found.


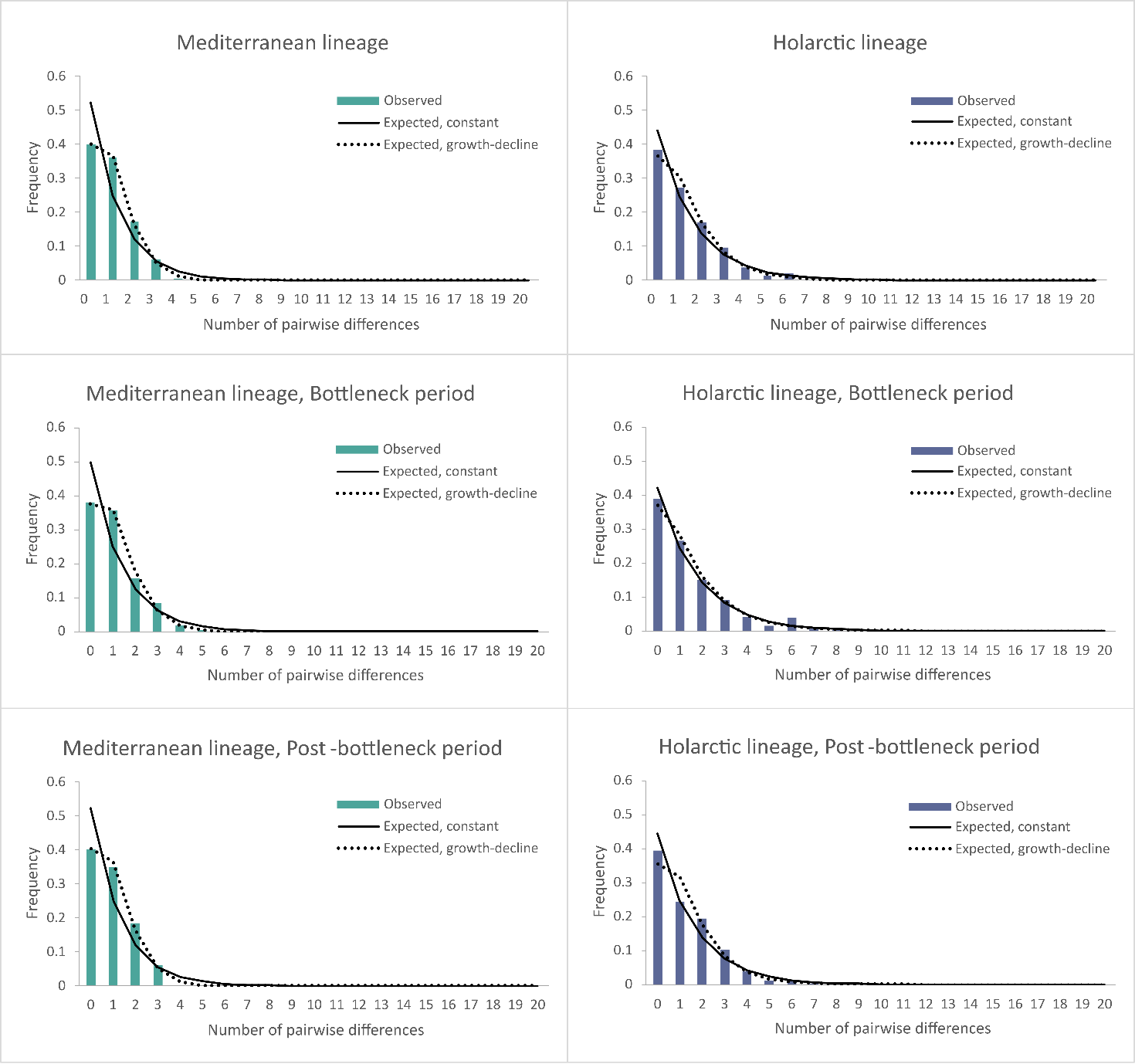


**Figure S12**. Mismatch distributions graphs for 393 Eurasian golden eagles from Mediterranean and Holarctic lineages, further split into temporal groups: Bottleneck and Post-bottleneck. The bars represent the observed allele frequencies, solid line – expected frequency under constant population size model, and dotted line – expected frequency under growth-decline model.

**Table S14.** The Bottleneck program analyses results. Wilcoxon’s sign rank test’s p-values (one-tail) for heterozygote excess and the mode-shift results of the distribution of allele frequencies. Asterisk indicates the result that suggests population bottleneck.

| **Group** | **Wilcoxon's test for heterozygote excess, p-value** | **Mode-shift** |
| --- | --- | --- |
| Northern Europe | 0.96 | normal L-shaped |
| Central and Eastern Europe | 0.78 | normal L-shaped |
| Central Asia and Caucasus | 0.85 | normal L-shaped |
| Far East | 0.28 | normal L-shaped |
| Mediterranean | 0.72 | shifted mode* |
| Holarctic | 0.96 | normal L-shaped |
| Bottleneck | 0.94 | normal L-shaped |
| Post-bottleneck | 0.71 | normal L-shaped |
| EURASIA | 0.97 | normal L-shaped |


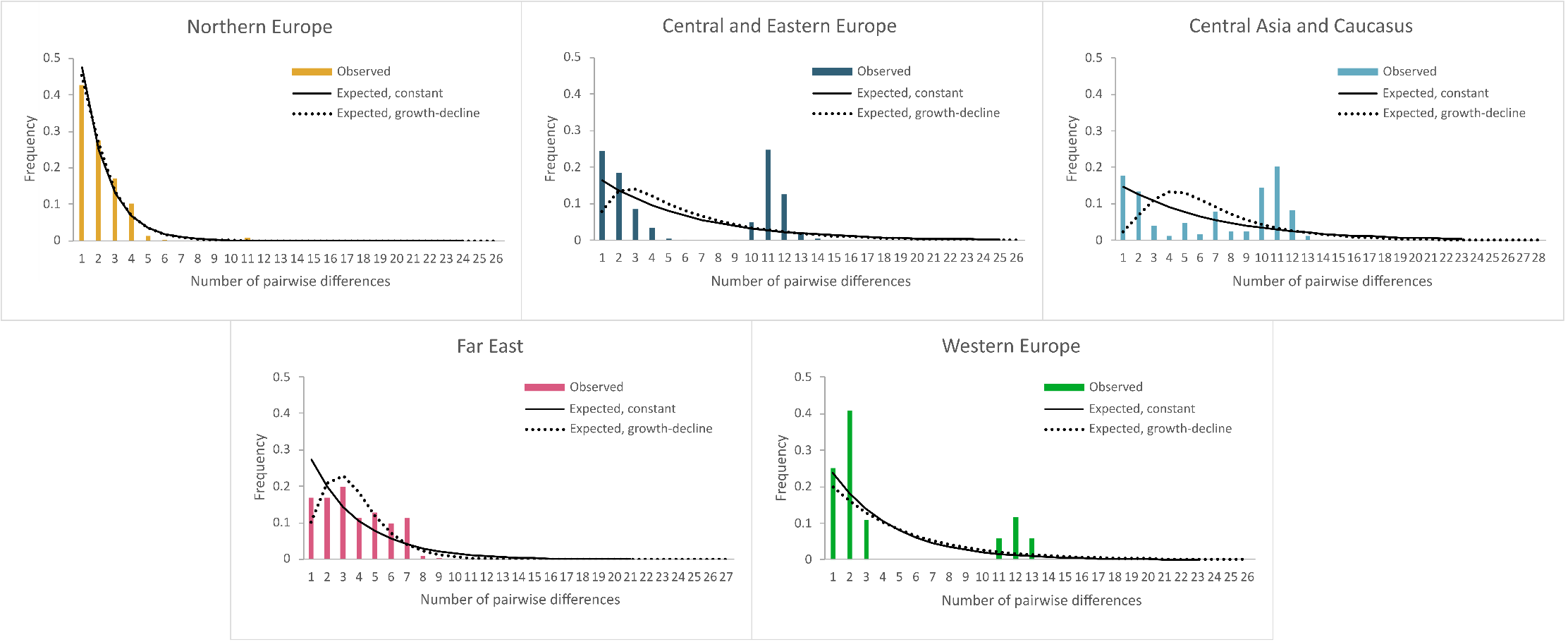


**Figure S13.** Mismatch distributions graphs for 434 Eurasian golden eagles from the five geographical groups: Northern Europe, Central and Eastern Europe, Central Asia and Caucasus, Far East, and Western Europe. The bars represent the observed allele frequencies, solid line – expected frequency under constant population size model, and dotted line – expected frequency under growth-decline model.


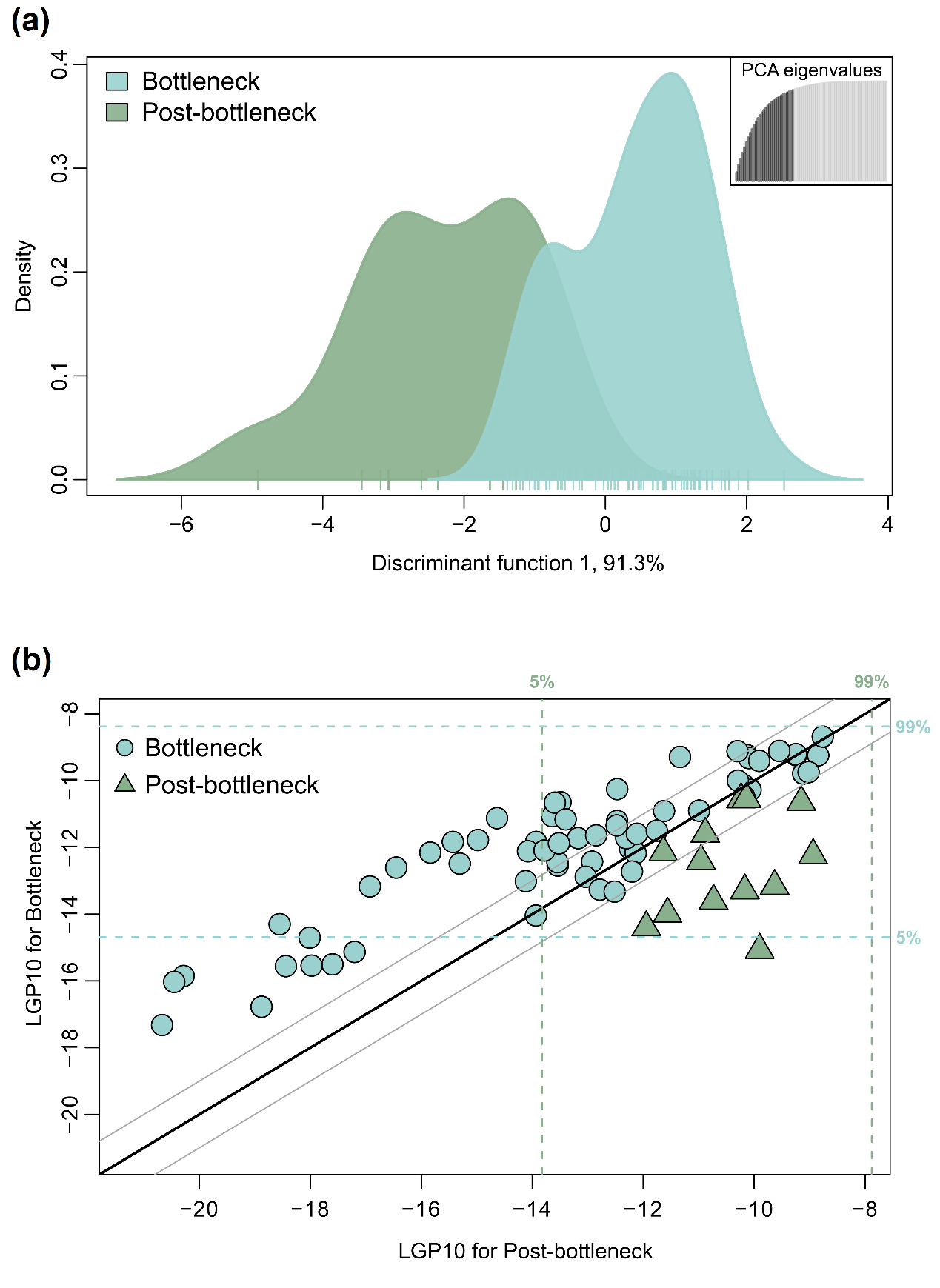


**Figure S14.** DAPC and GenePlot results for golden eagles from Bottleneck and Post-bottleneck groups. **(a)** DAPC plot that shows the first discriminant function and is based on the first 30 PCs that explain 91.3% of variation. **(b)** Log genotype probability (LGP) plot. The range where genetic assignment of individuals into these groups is likely, is outlined within 5% and 99% quantiles.


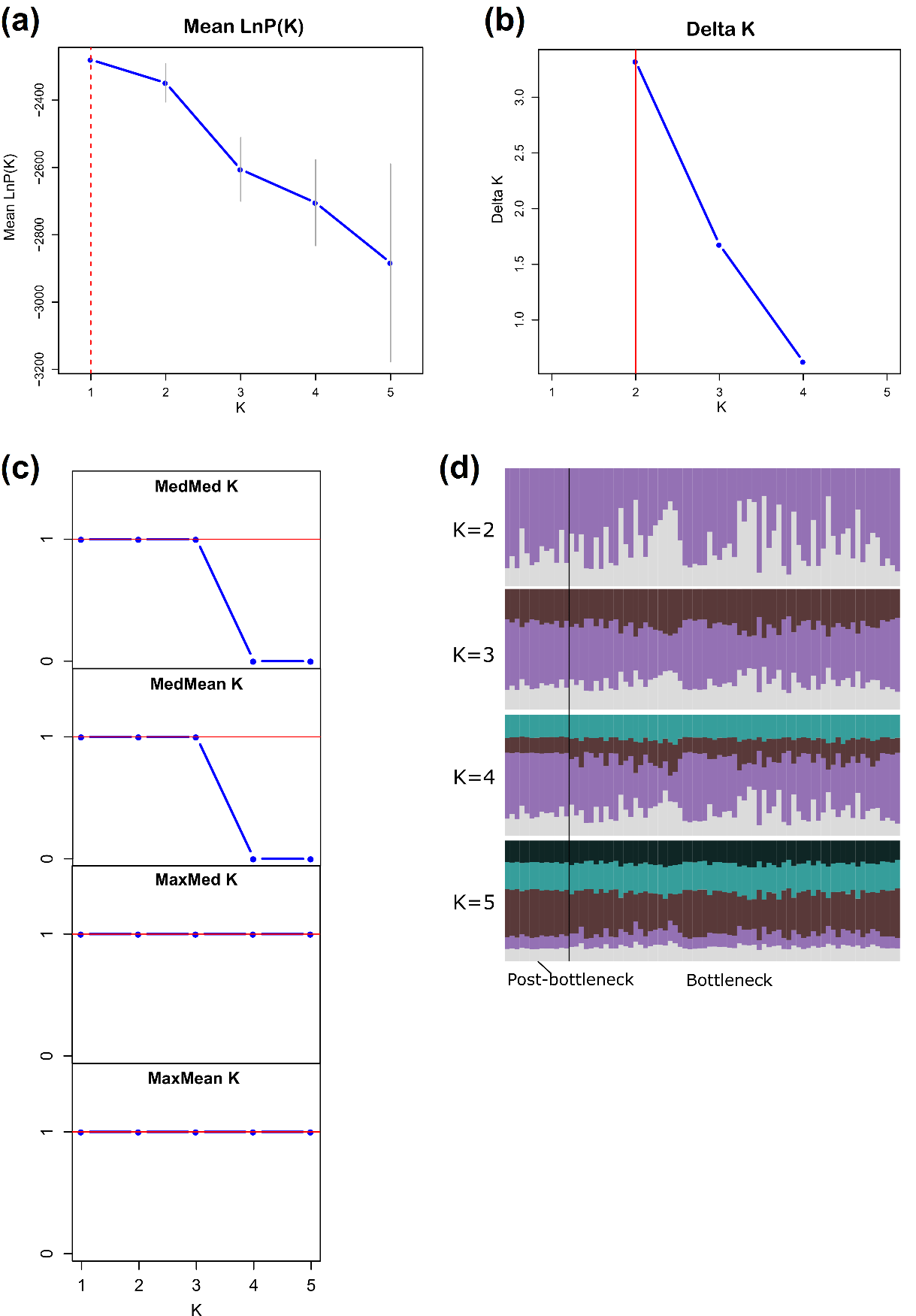


**Figure S15**. STRUCTURE results of cluster assignment of 80 golden eagles grouped according to the temporal groups (Bottleneck and Post-bottleneck) using 12 microsatellite loci for K = 1 to 5. **(a)** Log likelihood (L(K)) values for each tested K. **(b)** ΔK values (Evanno’s method) for each tested K. **(c)** Optimal K (Puechmaille method): median of the median (MedMed K), median of the mean (MedMean K), maximum of the median (MaxMed K), and maximum of the mean (MaxMean K) for each tested K. **(d)** STRUCTURE assignment plots for each tested K with temporal group as LOCPRIOR. Colors represent genetic clusters. Each individual is presented as a bar, and the amount of each color indicates the proportion of each inferred cluster.

**Table S15.** Pairwise ɸ_ST_ values for temporal groups of golden eagles across Eurasia, subdivided according to the mitochondrial lineage. The ɸ_ST_ were calculated with Kimura 2-parameter distance model. P-values after 10 000 permutations are in parentheses.

|  | **Bottleneck Holarctic** | **Bottleneck Mediterranean** | **Post-bottleneck Holarctic** |
| --- | --- | --- | --- |
| **Bottleneck Mediterranean** | 0.922  (p < 0.001) |  |  |
| **Post-bottleneck Holarctic** | -0.00242  (p = 0.632) | 0.929  (p < 0.001) |  |
| **Post-bottleneck Mediterranean** | 0.934  (p < 0.001) | 0.002  (p = 0.340) | 0.938  (p < 0.001) |

# **References**

Craig EH, Adams JR, Waits LP, *et al.* Nuclear and mitochondrial DNA analyses of golden eagles (Aquila chrysaetos canadensis) from three areas in western North America; initial results and conservation implications. *PLoS ONE* 2016; **11**.

Judkins ME**,** van den Bussche RA. Holarctic phylogeography of golden eagles (Aquila chrysaetos) and evaluation of alternative North American management approaches. *Biological Journal of the Linnean Society* 2017; **123**: 471–482.

Kylmänen A, Karabanina E, Ollila T, *et al.* Turnover and Natal Dispersal in the Finnish Golden Eagle (Aquila chrysaetos) Population. *Diversity* 2023; **15**: 567.

Nebel C, Gamauf A, Haring E, *et al.* Mitochondrial DNA analysis reveals Holarctic homogeneity and a distinct Mediterranean lineage in the Golden eagle (Aquila chrysaetos). *Biological Journal of the Linnean Society* 2015; **116**: 328–340.

Nebel C, Gamauf A, Haring E, *et al.* New insights into population structure of the European golden eagle (Aquila chrysaetos) revealed by microsatellite analysis. *Biological Journal of the Linnean Society* 2019; **128**: 611–631.

Ollila T. The Golden Eagle Aquila chrysaetos in Finland 2018. *Linnut-vuosikirja 2018* 2019: 104–109.

QGIS Development Team. QGIS Geographic Information System. 2022.

Sonsthagen SA, Coonan TJ, Latta BC, *et al.* Genetic diversity of a newly established population of golden eagles on the Channel Islands, California. *Biological Conservation* 2012; **146**: 116–122.

Sulkava S**,** Huhtala K**,** Rajala P. Diet and breeding success of the Golden Eagle in Finland 1958—82. *Annales Zoologici Fennici* 1984; **21**: 283–286.

Whitfield DP, Fielding AH, Mcleod DRA, *et al.* Modelling the effects of persecution on the population dynamics of golden eagles in Scotland. *Biological Conservation* 2004; **119**: 319–333.
